# The role of SWEET4 proteins in the post-phloem sugar transport pathway of *Setaria viridis* sink tissues

**DOI:** 10.1093/jxb/erad076

**Published:** 2023-03-08

**Authors:** Lily Chen, Diep R Ganguly, Sarah H Shafik, Florence Danila, Christopher P L Grof, Robert E Sharwood, Robert T Furbank

**Affiliations:** Research School of Biology, ARC Centre of Excellence for Translational Photosynthesis, Australian National University, Canberra, Australian Capital Territory 2601, Australia; Hawkesbury Institute for the Environment, Western Sydney University, Hawkesbury Campus, New South Wales 2753, Australia; Research School of Biology, ARC Centre of Excellence in Plant Energy Biology, Australian National University, Canberra, Australian Capital Territory 2601, Australia; CSIRO Synthetic Biology Future Science Platform, Canberra, Australian Capital Territory 2601, Australia; Research School of Biology, Australian National University, Canberra, Australian Capital Territory 2601, Australia; Research School of Biology, ARC Centre of Excellence for Translational Photosynthesis, Australian National University, Canberra, Australian Capital Territory 2601, Australia; Centre for Plant Science, School of Environmental and Life Sciences, College of Engineering Science and Environment, University of Newcastle, Callaghan, New South Wales 2308, Australia; Hawkesbury Institute for the Environment, Western Sydney University, Hawkesbury Campus, New South Wales 2753, Australia; Research School of Biology, ARC Centre of Excellence for Translational Photosynthesis, Australian National University, Canberra, Australian Capital Territory 2601, Australia; Northwest Agriculture and Forestry University, China

**Keywords:** Apoplast, glucose transporter, grasses, seed, Setaria, sucrose transporter, sugar transport, SWEET

## Abstract

In the developing seeds of all higher plants, filial cells are symplastically isolated from the maternal tissue supplying photosynthate to the reproductive structure. Photoassimilates must be transported apoplastically, crossing several membrane barriers, a process facilitated by sugar transporters. Sugars Will Eventually be Exported Transporters (SWEETs) have been proposed to play a crucial role in apoplastic sugar transport during phloem unloading and the post-phloem pathway in sink tissues. Evidence for this is presented here for developing seeds of the C_4_ model grass *Setaria viridis*. Using immunolocalization, SvSWEET4 was detected in various maternal and filial tissues within the seed along the sugar transport pathway, in the vascular parenchyma of the pedicel, and in the xylem parenchyma of the stem. Expression of *SvSWEET4a* in *Xenopus laevis* oocytes indicated that it functions as a high-capacity glucose and sucrose transporter. Carbohydrate and transcriptional profiling of Setaria seed heads showed that there were some developmental shifts in hexose and sucrose content and consistent expression of *SvSWEET4* homologues. Collectively, these results provide evidence for the involvement of SWEETs in the apoplastic transport pathway of sink tissues and allow a pathway for post-phloem sugar transport into the seed to be proposed.

## Teaser

The sucrose and glucose transporter SWEET4 was localized to the seed and stem of *Setaria viridis*, indicating a role in facilitating sugar supply to carbon sink tissues.

## Introduction

Photosynthate transport to and within the developing seed of cereals is complex, since filial cells are symplastically isolated from maternal cells (i.e. their cytoplasms are not connected by plasmodesmatal pores), and considerable diversity exists in the post-phloem pathway of sugar movement in seeds of higher plants (Thorne, 1985; Patrick and Offler, 1995). The seed/grain is generally the most agronomically valuable part of a crop, and yield is strongly correlated with sink-located transport and transfer processes (Gifford and Evans, 1981). Seeds have also been a widely used model for sugar movement into sinks as they are morphologically discrete entities, can be readily manipulated experimentally, and are committed to the storage of carbohydrates (Thorne, 1985; Patrick and Offler, 1995).

Seed development in plants can generally be divided into four stages, beginning with cell division, then expansion, differentiation, and storage (Evers and Millar, 2002). Within cereal seeds, these shifts have been linked to high invertase activity, which results in high hexose to sucrose ratios during cell division (Cheng and Chourey, 1999; Weschke *et al.*, 2003). Later in development when cell expansion and storage begins, this changes to a high sucrose to hexose ratio, concomitant with high sucrose synthase (SuSy) activity (Borisjuk *et al.*, 2004). The seed is broadly comprised of the pericarp/seed coat, nucellus, embryo and endosperm, separated by the scutellum (Evers and Millar, 2002). While the general structures of monocot and dicot seeds are somewhat similar, there are some stark differences between species. Cereals are known for large starchy seeds, unlike dicots such as Arabidopsis (*Arabidopsis thaliana*), and hence require a large flux of photoassimilates to occur during seed filling.

The partitioning of photoassimilates between the vegetative tissues and the agronomically valuable grain has a large impact on yield (Thorne, 1985). Following anthesis, the seed or grain becomes the predominant sink for carbohydrates, especially in monocots (Palmer *et al.*, 1973). Generally, in crop seeds, sugars are unloaded symplastically via plasmodesmatal connections between one or more maternal cells and the phloem before entering the apoplast surrounding filial tissues. In developing seeds, filial tissue, such as the embryo and endosperm, is symplastically isolated from maternal tissues such as the seed coat and nucellar cells soon after fertilization (Patrick, 2012). Subsequently, to support the growth and development of the embryo and the large endosperm in monocots, sugars must be transferred via an apoplastic pathway across plasma membranes, thus requiring membrane-localized sugar transporters. Transporters must facilitate not only the export of sugars from the maternal cells into the apoplast but also subsequent uptake into the filial cells (Patrick and Offler, 1995; Patrick, 2012).

In monocots, the active sucrose transporter SUT1 has long been implicated in seed filling, functioning to import sucrose from the apoplast surrounding seed maternal tissues into filial cells. Evidence has shown that SUT1 transcript and protein were abundant in cereal seeds, localized to cells at the interface between maternal and filial tissues, and that suppression or knockout of this transporter resulted in highly impaired grain filling (Bagnall *et al.*, 2000; Weschke *et al.*, 2000; Furbank *et al.*, 2001). For example, antisense suppression of SUT1 in rice resulted in little to no grain filling, leading to a shrivelled grain phenotype (Scofield *et al.*, 2002). Until recently, the mechanism of sugar export out of maternal cells into the apoplast for uptake by SUTs has remained elusive. The discovery of Sugars Will Eventually be Exported Transporters (SWEETs) potentially provided a mechanism for this process, with recent studies suggesting that SWEETs first export sugars into the apoplast before being imported into cells by SUTs or hexose transporters (HXTs) depending on the nature of sugars present at the membrane interface (Chen *et al.*, 2015; Sosso *et al.*, 2015; Ma *et al.*, 2017; Yang *et al.*, 2018; Doll *et al.*, 2020). Despite this potentially universal model for SWEET function, the diversity in seed morphology and structure present even in the grasses means that many components of sugar movement during seed filling remain unresolved (Cochrane and Duffus, 1979). Numerous post-phloem membrane barriers exist within a single seed, thus SWEETs could potentially be found at various interfaces. Previous studies using reverse genetics which have implicated SWEETs in this transport pathway (Sosso *et al.*, 2015) have not resolved this issue and prevent the elucidation of a model pathway for post-phloem sugar transport which includes the involvement of SWEETs.

The genus Setaria contains a number of small grain millets, notably *Setaria italica* (Foxtail millet), one of the oldest domesticated and widely adapted grain crops globally and an important component of food production in developing countries (Huang *et al.*, 2016). *Setaria viridis* (Green foxtail), a small, closely related, easily transformable relative with a small sequenced genome, is becoming a widely used genetic model for functional genomics and genetics in millets (Brutnell *et al.*, 2010). Despite the importance of millets, the pathway of post-phloem sugar movement in their grains is poorly characterized. Here we seek to understand the role SWEETs might play in phloem unloading and the post-phloem pathway during seed development and filling in *S. viridis* (Setaria). Previous literature has seen a trend in *SWEET4* orthologues being expressed predominantly in sinks such as stems and/or seeds of sorghum, rice, maize, sugarcane, and Setaria, suggesting a role in phloem unloading or the steps immediately following this (Sosso *et al.*, 2015; Martin *et al.*, 2016; Mizuno *et al.*, 2016; Hu *et al.*, 2018). Here SWEET4a from *S*. *viridis* is examined with a particular focus on the seed head using a combination of immunohistochemistry, functional characterization, major carbohydrate profiling, and expression analyses. These results expand our understanding of SWEETs in the phloem unloading and post-phloem pathway of C_4_ grasses, and the data are used to propose a pathway for sugar movement from the phloem to the endosperm tissue of Setaria seeds.

## Materials and methods

### Plant growth and harvesting

*Setaria viridis* A.10 and ME034V was grown in a PGC Flex growth chamber (Conviron, Winnipeg, Canada). The day/night lengths and temperatures were 16 h/8 h and 28 °C/20 °C, respectively, with 55% humidity and light at an intensity of ~600 μmol m^−2^ s^−1^ supplied by halogen incandescent lamps (42 W 2800K warm white clear glass 630 lumens, CLA, Brookvale, NSW, Australia) and Pentron Hg 4 ft fluorescent tubes (54 W 4100K cool white, Sylvania, Wilmington, MA, USA). Plants were grown in 1.7 litre pots filled to 90% capacity using Green Wizard Premium Potting mix (Debco, Bella Vista, NSW, Australia), and the remaining 10% filled with Debco Seed Raising and Superior Germinating Mix, both supplemented with 1 g kg^–1^ of slow-release fertilizer (Osmocote, Scotts, Bella Vista, NSW, Australia). As the seed itself is very small in Setaria, whole seed heads were harvested at the base of each from four developmental stages: 50% seed head emergence, anthesis, 8 days after anthesis (DAA), and 16 DAA (Supplementary Fig. S1). Each stage has four biological replicates where each biological replicate is made up of four individual plants. Samples were harvested into 10 ml vials and immediately snap-frozen in liquid nitrogen. Samples were homogenized cryogenically with the TissueLyser II (QIAGEN, Hilden, Germany).

### Plant protein extraction

Samples were harvested from plants at the 50% seed head emergence stage. Organs sampled were the youngest fully expanded leaf, stem, and seed head. Protein was extracted using the Plant Fractionated Protein Extraction Kit (Sigma-Aldrich, St. Louis, MO, USA) from 200 mg of cryogenically homogenized plant tissue according to the manufacturer’s instructions. Protein extractions were quantified using a Bradford assay (ThermoFisher Scientific, Waltham, MA, USA) adapted from Bradford (1976). The purified rice RbcL protein was kindly donated by Timothy Rhodes and isolated through size exclusion chromatography as described previously (Sharwood *et al.*, 2008). Samples were supplemented with NuPAGE™ lithium dodecyl sulfate buffer (LDS, 4×, ThermoFisher Scientific).

### Immunoblotting

Protein extracts in LDS were supplemented with 10% β-mercaptoethanol (v/v) immediately before use. Samples were heated at 37 °C for 15 min since membrane proteins tend to aggregate at higher temperatures. Samples were spun down and loaded onto 4–12% Bis-Tris acrylamide SDS–PAGE protein gels (ThermoFisher Scientific). Protein gels were first run for 15 min at 140 V and then at 200 V for a further 30 min at 4 °C in a running buffer (25 mM MES, 25 mM Tris, 1.7 mM SDS, 400 μM EDTA-Na_2_). After separation with SDS–PAGE, proteins were transferred to a polyvinylidene fluoride (PVDF) membrane using the XCell II™ Blot Module (ThermoFisher Scientific) in transfer buffer [25 mM Bicine, 25 mM Bis-Tris, 1 mM EDTA, 10% (v/v) methanol]. Following the transfer, the membrane was blocked overnight at 4 °C in Tris-buffered saline+Tween-20 [TBST; 5 mM Tris, 15 mM NaCl, 0.1% (v/v) Tween-20] supplemented with 5% (w/v) skim milk powder and 3% (w/v) BSA. Antibody peptides were designed and commercially synthesized to create the antigen-purified SvSWEET4 antiserum [GL Biochem (Shanghai) Ltd, Shanghai, China, http://www.glbiochem.com/en/index/index.aspx]. The tobacco RbcL antiserum was kindly donated by Professor Spencer Whitney (Whitney *et al.*, 2001). SvSWEET4 antiserum was diluted to 1:3000 in TBST+5% (w/v) skim milk powder or 1:10 000 for the tobacco RbcL antiserum, added to the membrane, and incubated on an orbital rotating platform at room temperature (22–24 °C) for 60 min. This was followed by 3 × 10 min washes with TBST. Goat anti-rabbit horseradish peroxidase-conjugated secondary antibody (Cat: 1706515; Bio-Rad, Hercules, CA, USA) diluted to 1:10 000 in TBST+5% (w/v) skim milk powder was added to the membrane and incubated for 60 min at room temperature (22–24 °C) on an orbital rotating platform. The membrane was again washed for 3 × 10 min in TBST. Membranes were developed by addition of the Western Lightning Plus enhanced chemiluminescent reagent (Cat: NEL103E001EA; PerkinElmer, Waltham, MA, USA) and imaged on the ChemiDoc™ Gel Imaging System (Bio-Rad).

### Immunolocalization of SvSWEET4

Fresh seeds were harvested into H_2_O from ~16 DAA plants. Thin hand sections were blocked in a 96-well plate for 2 h in 20 μl of TBST [50 mM Tris, 150 mM NaCl, 0.1% (v/v) Tween-20], +3% skim milk powder (w/v) and then incubated overnight in primary antiserum diluted 1:40 with blocking solution. Following the incubation, sections were washed 6 × 10 min in blocking solution. Antibodies against plasma membrane intrinsic proteins (PIPs) aquaporins (PIP1;1, PIP1;2, PIP1;3, PIP1;4, and PIP1;5; Aquaporins Cat: AS09487; Agrisera, Vännäs, Sweden) were used as positive controls. Since the SvSWEET4 antiserum was antigen purified, the undiluted pre-immune serum could not be used as a negative control due to the greatly reduced immunoglobulin concentration of the purified serum. Therefore, a protein quantification was carried out using a Bradford assay as outlined previously. From here, the pre-immune serum was diluted to approximately the same concentration as the antibodies for use as a negative control. The positive and negative controls were treated in the same way as the samples probed with SvSWEET antiserum prior to their incubation with secondary antibody. Sections were then incubated with the secondary goat anti-rabbit IgG (H+L) Alexa Fluor Plus 488-conjugated antibody (Cat: A-11001; ThermoFisher Scientific) diluted 1:200 with the blocking solution. Sections were again washed 6 × 10 min with the blocking solution, stained with Calcofluor white (0.05%, v/v) to visualize the cell walls, and then washed with distilled water. Sections were mounted onto microscope slides with 50% glycerol. Images were taken on the confocal microscope Zeiss Axio Imager 2 (Zeiss, Jena, Germany, https://www.zeiss.com). The channels that were used were as follows: fluorescein isothiocyanate (FITC; ex 493 nm, em 517 nm), and UV for cell walls (ex 254 nm, em 432 nm). The images were processed with ZEN Blue 3.1 (Zeiss).

### *SvSWEET4a* cRNA synthesis

The coding sequence (CDS) of *SvSWEET4a* was commercially synthesized (GenScript, Piscataway, NJ, USA) and ligated into the PENTR1A-GW vector. This vector harbouring the gene of interest was recombined into the destination vector pGEMHE-GW using LR clonase (Cat: 11791020; ThermoFisher Scientific). The expression vectors were linearized with the restriction enzyme *Nhe*I-HF (Cat: R3131S; New England Biolabs, Notting Hill, VIC, Australia) overnight at 37 °C and then incubated at 65 °C for 15 min for enzyme inactivation. The DNA was cleaned up using the MinElute Reaction Cleanup Kit (QIAGEN) and resuspended in 12 μl of RNase-free H_2_O. A 1 μg aliquot of linearized expression vector was transcribed to capped RNA (cRNA) using the T7 Ambion mMessage mMachine kit according to the manufacturer’s instructions (ThermoFisher Scientific). The cRNA was supplemented with ammonium acetate stop solution (5 M ammonium acetate, 100 mM EDTA; supplied in the kit) and treated with a phenol–chloroform clean-up as detailed by the manufacturer (ThermoFisher Scientific). The final cRNA was resuspended in 22 μl of RNase-free H_2_O and checked for the presence of a single band on an agarose gel.

### *Xenopus laevis* frogs

Ethical approval of the work performed with female Xenopus frogs was obtained from the Australian National University (ANU) Animal Experimentation Ethics Committee (Animal Ethics Protocol Numbers A2019/26) in accordance with the Australian Code of Practice for the Care and Use of Animals for Scientific Purposes. The frogs were purchased from Nasco (catalogue no. LM00535M) and were housed in the Xenopus Frog Facility of the ANU Research School of Biology in compliance with the relevant institutional and Australian Government regulations.

### Isolation and injection of Xenopus oocytes

Adult female frogs were anaesthetized in a solution of ethyl 3-aminobenzoate methanesulfonate salt (1.6 g l^–1^) and 1 mM NaHCO_3_ using a method described previously (van Schalkwyk *et al.*, 2015). Briefly, sections of the ovary were removed and the collagenous membrane encasing the oocytes was degraded by collagenase B (Cat: COLLB-RO, Sigma-Aldrich), the amount of which was dependent on the harvest size. The collagenase-treated oocytes were washed 10 times with ‘calcium-free oocyte ringer’ buffer (OR^2–^; 82.5 mM NaCl, 2.5 mM KCl, 1 mM MgCl_2_, 1 mM Na_2_HPO_4_, 5 mM HEPES; pH 7.8), five times with ‘calcium-containing oocyte ringer’ (OR^2+^) buffer (OR^2–^ buffer supplemented with 1 mM CaCl_2_ and 50 μg ml^–1^ penicillin and streptomycin), and then were incubated in L-15 (Leibovitz) medium with l-glutamine (Cat: L1518; Sigma-Aldrich) supplemented with 10 mM HEPES and 50 μg ml^–1^ penicillin and streptomycin. Stage V–VI oocytes were microinjected with cRNA encoding *SvSWEET4a*, *SvSWEET13a*, or *SvSWEET13b* (15 ng per oocyte, unless specified otherwise). In a subset of experiments that were performed to optimize the amount of cRNA required for *SvSWEET4* expression, oocytes were microinjected with cRNA amounts ranging between 2.5 ng and 25 ng. Oocytes were stored at 16–18 °C in OR^2+^ buffer supplemented with 50 μg ml^–1^ penicillin and streptomycin. The approximate optimum cRNA amount and incubation time, for maximum uptake (i.e. highest signal to noise), was determined for each sugar prior to transport kinetics (Supplementary Fig. S2, glucose, 90 min; sucrose, 30 min).

### Oocyte protein extraction for immunoblotting

The detection of SvSWEET protein in the oocyte membrane was achieved using oocytes 3 d post-cRNA injection as previously described (Summers *et al.*, 2014). Briefly, 20 oocytes expressing either *SvSWEET4a*, *SvSWEET13a*, or *SvSWEET13b* were homogenized in 1 ml of 20 mM Tris–HCl (pH 8) supplemented with cOmplete™ mini EDTA-free protease inhibitor cocktail (Roche, Basel, Switzerland). The homogenate was centrifuged at 1000 *g* for 10 min at 4 °C, and the supernatant was transferred to a new tube and mixed by inversion. A 37 μl aliquot was diluted in 400 μl of 20 mM Tris–HCl (pH 8.0) and centrifuged at 21 000 *g* for 20 min at 4 °C. The supernatant was removed, and the pellet was washed with 1 ml of a buffer containing 20 mM Tris–HCl (pH 8) and 1 M NaCl supplemented with the protease inhibitor cocktail, and was centrifuged at 21 000 *g* for 10 min at 4 °C. The pellet was solubilized in 25 μl of a solution comprising 1% (v/v) Triton X-100, 20 mM Tris–HCl (pH 8), 10 mM NaCl, and 25% (v/v) NuPage LDS (4×, ThermoFisher Scientific) to create non-reducing conditions. Another set of samples were prepared where the pellets were solubilized in 25 μl of a solution comprising 1% (v/v) Triton X-100, 20 mM Tris–HCl (pH 8), 10 mM NaCl, 25% (v/v) NuPage LDS (4×, ThermoFisher Scientific), 150 mM DTT, and 10% (v/v) β-mercaptoethanol to create reducing conditions. The proteins present in the oocyte membrane preparations were run on 4–12% Bis-Tris acrylamide SDS–PAGE protein gels (ThermoFisher Scientific), and then immunoblotting was performed as described in the previous section.

### Transport assays

Transport assays were performed as in L. Chen *et al.* (2022). The radiolabelled sugars were purchased from American Radiolabelled Chemicals ([^3^H]glucose, 60 Ci mmol^–1^; [^14^C]sucrose, 0.6 Ci mmol^–1^) (ARC, St. Louis, MO, USA). The SvSWEET4a transport assays were performed on days 3–4 post-cRNA injection and, unless specified otherwise, were conducted over 90 min for the [^3^H]glucose uptake assays and 30 min for the [^14^C]sucrose uptake assays. The transport assays were conducted at 27.5 °C in ND96 buffer (96 mM NaCl, 2 mM KCl, 1 mM MgCl_2_, 1.8 mM CaCl_2_, 10 mM MES, and 10 mM Tris-base; pH 7.4). Transmembrane domain predictions indicate that the N- and C-terminus of SvSWEET4a, as well as other previously published SWEETs, are on the extracellular side and cytosolic side, respectively, and thus, are predicted to adopt the same orientation in the oocyte plasma membrane (Chen *et al.*, 2010). In all cases, at least three independent experiments were performed (on different days and using oocytes from different frogs) and, within each experiment, measurements were made from 8–10 oocytes per treatment. The oocytes were transferred to a 5 ml polystyrene round bottom tube (*In Vitro* Technologies, Lane Cove West, NSW, Australia) and washed twice with 3.5 ml of ND96 buffer (pH 7.4), and the residual solution was removed by pipette. To commence the assay, the oocytes were suspended in 100 μl of ND96 buffer supplemented with either [^3^H]glucose and unlabelled glucose or [^14^C]sucrose and unlabelled sucrose. Analyses of the kinetics of glucose transport via SvSWEET4a were undertaken by measuring the uptake of [^3^H]glucose (400.8 μM) in the presence of 0–175 mM unlabelled glucose. Likewise, the kinetics of sucrose transport via SvSWEET4a were determined by measuring the uptake of [^14^C]sucrose (668 μM) in the presence of 0–175 mM unlabelled sucrose. In all cases, the incubation was terminated by removing the reaction buffer and washing the oocytes twice with 3.5 ml of ice-cold ND96 pH 7.4 buffer. Each oocyte was then transferred to a well of a white 96-well plate, lysed by an overnight incubation at room temperature (22–24°C) with 20 μl of 5% (w/v) SDS, and then mixed with 150 μl of MicroScint-40 microscintillant (PerkinElmer). The plate was covered with a TopSeal-A (PerkinElmer) and the radioactivity within each well measured with a MicroBeta^2^ microplate liquid scintillation analyzer (PerkinElmer). The component of transport attributable to SvSWEET4a (i.e. SvSWEET-mediated transport) was calculated by subtracting the ‘background’ level of [^3^H]glucose or [^14^C]sucrose accumulation detected in the non-expressing oocytes from that measured in oocytes expressing SvSWEET4a. The kinetic parameters for glucose and sucrose transport via SvSWEET4a were determined by a least-squares fit of the Michaelis–Menten equation to the data using GraphPad Prism version 8.0.0 for Windows (GraphPad Software, San Diego, CA, USA, https://www.graphpad.com).

### Enzyme-coupled quantification of carbohydrates

Soluble and insoluble sugars were measured from 20 mg of homogenized plant tissue and extracted as outlined by Stitt *et al.* (1989). Two technical replicates were performed for this assay where each replicate contained four biological replicates. To quantify soluble sugars (glucose, fructose, sucrose, and fructans), an ethanolic extraction was performed on the tissue. Enzyme-coupled assays were performed using hexokinase, phosphoglucose isomerase, sucrase, and endo- and exo-inulinase (fructanase) to measure glucose, fructose, sucrose, and fructans, respectively. This method is based on quantitative reduction of NADP^+^ and spectrophotometric detection of NADPH at 340 nm. Following the ethanolic extraction of soluble sugars, the remaining starch pellet was first hydrolysed and then enzymatically digested using α-amylase and amyloglucosidase. This enzymatic method uses hexokinase and is based on the degradation of starch to glucose units. As with soluble sugars, the reduction of NADP^+^ to NADPH was measured spectrophotometrically at 340 nm. These methods were thoroughly detailed previously (L. Chen *et al.*, 2022).

### mRNA sequencing

Total RNA was extracted from 150 mg of frozen tissue using the Aurum™ Total RNA Fatty and Fibrous Tissue Kit (Bio-Rad) according to the manufacturer’s instructions. RNA concentrations, and 230/260 and 260/280 ratios were checked on a NanoDrop™ 3300 Fluorospectrometer (ThermoFisher Scientific). Samples were sent on dry ice to The Next Generation Sequencing Facility, Hawkesbury Campus, Richmond, Western Sydney University. cDNA libraries were prepared at the facility using the TruSeq RNA Library Prep Kit v2 with poly(A) RNA enrichment (Illumina, San Diego, CA, USA, https://www.illumina.com/). The libraries were run through the HiSeq 2500 System (high-output) over two lanes with 2 × 125 bp read lengths. Raw read quality was diagnosed using FastQC (v0.11.2). Trim Galore! (v0.6.5) was used to trim adaptors and low-quality base calls with PHRED <20 (–q 20) followed by further inspection with FastQC. Trimmed reads were employed for paired-end alignments using Subjunc, from the Subread package (v1.6.2, Liao *et al.*, 2013), considering uniquely mapped reads only, which were then sorted and indexed using Samtools (v1.2, Li *et al.*, 2009). Transcript quantification was performed using featureCounts (–s 0 for non-stranded quantification, Liao *et al.*, 2014) at the gene level based on the *S. viridis* v2.1 genome annotation (Bennetzen *et al.*, 2012), which was obtained from Phytozome v12 (http://phytozome.jgi.doe.gov/). Raw counts were then imported into edgeR for loci detected at counts per million (CPM) >1 in at least three samples (Robinson *et al.*, 2009). Library normalization was performed using the trimmed mean of M-values (TMM) method to account for sequencing depth and composition (Robinson and Oshlack, 2010). TMM normalized counts were converted into log_2_ fragments per kilobase of transcript per million mapped reads (FPKM), using the fpkm function and annotated gene lengths, then visualized using the R function pheatmap. The code used for analyses are available on GitHub: https://github.com/dtrain16/NGS-scripts. A summary of sequencing statistics and log2 FPKM values for genes of interest can be found in Supplementary Dataset S1 and Supplementary Fig. S3.

### Quantitative PCR (qPCR) analysis

In the qPCR analysis, four biological replicates pooled from three plants per replicate were used for each tissue sampled. RNA was extracted as described above and treated with DNase to exclude genomic DNA contamination. RNA concentrations, and 230/260 and 260/280 ratios were checked on a NanoDrop™ 3300 Fluorospectrometer (ThermoFisher Scientific). A 1 µg aliquot of RNA was used to create cDNA according to the manufacturer’s instructions using the SuperScript™ III First-Strand Synthesis System (ThermoFisher Scientific). A cDNA dilution of 1:100 was used for qPCR, except for *SvSWEET1b* where a dilution of 1:25 was used. Transcript levels were quantified using the ΔCt method based on the values given by the qPCR data from three technical replicates (Vandesompele *et al.*, 2002). Transcript levels were given relative to the reference genes. *Elongation factor 1-α* and *translation factor* were used as reference genes in this analysis. The primers used are outlined in Supplementary Table S1.

### Statistical analysis

The statistical tests were performed using GraphPad Prism version 8.0.0. A one-way ANOVA followed by Tukey’s multiple comparisons test was performed for each carbohydrate profile to detect significant differences between leaf sections. For the transport assays, an unpaired Student’s *t*-test was performed. All errors cited in the text and shown in the figures represent the SEM. Significance was defined as *P*<0.05.

## Results

### Immunodetection of SvSWEET4 in the seed and stem tissue

To investigate the role of SvSWEET4 in the sink tissues, the localization of the native protein was first examined in the seed heads and stems, and so antibodies were raised against peptides derived from the C-terminus of SvSWEET4a (Supplementary Fig. S4, <30% sequence similarity within the C-terminal tail between the SvSWEETs). While this region is specific to the SvSWEET4a protein, it is highly homologous to the other SvSWEET4 proteins; therefore, it is possible that it does not distinguish between isoforms and therefore will be referred to as ‘SvSWEET4 antiserum’ (Supplementary Figs S4, S5A, B). This could not have been avoided due to high sequence similarity not only of the protein sequence but also of the CDS (>95%) (Supplementary Fig. S5E). This is also apparent in other species such as maize and sorghum which also have three isoforms (Supplementary Fig. S5). There was low sequence similarity in the epitope regions used across all other SvSWEETs.

**Fig. 1.**
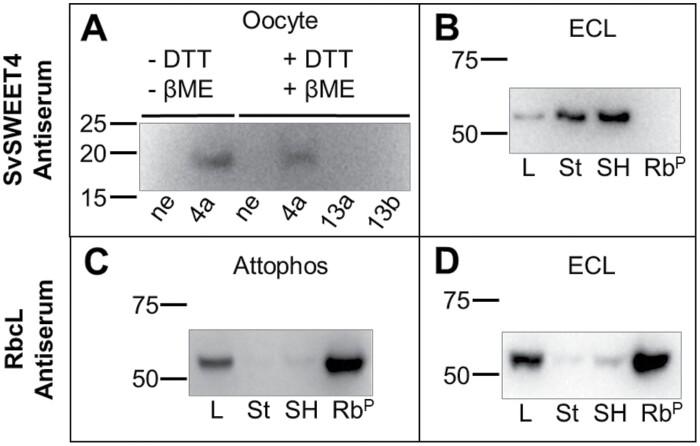
Validation of SvSWEET4 antiserum using immunoblotting. Commercially generated affinity-purified antiserum was raised against peptides derived from the SvSWEET4a protein isoform (see Supplementary Figs S4 and S5). This was tested for specificity for SvSWEET4 against total protein extracts from Xenopus oocytes expressing SvSWEET4a, SvSWEETT13a, or SvSWEET13b (A) and total plant protein extracts from Setaria (B–D). In (A) the exclusion or addition (–/+) of reducing agents dithiothreitol (DTT) and β-mercaptoethanol (βME) is indicated above the blot. The lysates from non-expressing (ne), SvSWEET13a-, and SvSWEET13b-expressing oocytes were used as negative controls. Recombinant protein was isolated and loaded from 20 Xenopus oocytes. Total plant extracts from the leaf (L), stem (St), and seed head (SH) were pooled from 12 Setaria plants harvested at 50% seed head emergence stage and 10 µg of protein was loaded. Purified Rubisco large subunit (Rb^P^) from rice was used as a control and 4 µg was loaded. (A), (B), and (D) used electrochemiluminescence (ECL) to detect the horseradish peroxidase (HRP) secondary antibody conjugate. (C) The membrane from (B) probed for the Rubisco large subunit (RbcL) using antiserum derived from tobacco RbcL peptides and the use of the AttoPhos substrate system to detect alkaline phosphatase (AP) secondary antibody conjugate. RbcL was probed for using a separate membrane in (D) using the same method as in (B). Molecular weight standards (kDa) are indicated to the left of each blot. Uncropped immunoblots are shown in Supplementary Fig. S6.

The specificity of the antiserum for SvSWEET4 was tested using total protein extracts from Xenopus oocytes, expressing recombinant SvSWEET4a, SvSWEET13a, and SvSWEET13b, or Setaria tissue (Fig. 1; Supplementary Fig. S6). Oocyte lysates were treated with or without the reducing agents DTT and β-mercaptoethanol to test for dimerization. For recombinant protein expressed in oocytes, the monomer form at ~20 kDa was present regardless of whether reducing agents were added. There was no cross-reactivity with non-expressing, SvSWEET13a or SvSWEET13b oocyte lysate. Having established functional and SvSWEET4-specific antiserum using *in vitro* assays, SvSWEET4 antiserum was also tested on total plant protein extracts from the leaf, stem, and seed head for specificity (Fig. 1B). Purified Rubisco large subunit (RbcL) from rice (94.1% similarity to Setaria) was used as a control for cross-reactivity. SvSWEET4 was detected as a putative dimer at ~55 kDa. This is in accord with the predicted monomer size of SvSWEET4 orthologues of ~27 kDa, and dimerization of SvSWEET4 proteins is consistent with previous studies on the structural analysis of eukaryotic SWEETs (Tao *et al.*, 2015). The SvSWEET4 dimer recognized from plant tissue has the same approximate size as RbcL (~50–55 kDa) (Whitney *et al.*, 2001). Given the abundance of this protein in all green tissues, to rule out the possibility that SWEET4 antiserum was cross-reacting with RbcL, further testing was conducted. The same membrane used in Fig. 1B was probed again but using RbcL antiserum derived from peptides made to the tobacco RbcL to ensure the SvSWEET4 antiserum was not cross-reacting with Rubsico (Fig. 1C). The leaf tissue and purified rice RbcL samples gave a strong signal, as expected, when probed with the RbcL antiserum (Fig. 1C, D), but a very weak signal when probed with the SvSWEET4 antiserum (Fig. 1B). Conversely, the sink tissues comprising stem and the seed head, which showed a strong signal with SWEET4 antiserum, resulted in very weak signals when protein was probed with the RbcL antiserum (Fig. 1C). Cumulatively these results demonstrate that the SvSWEET4 antiserum is not cross-reacting with Rubisco and is specific for SvSWEET4 proteins.

**Fig. 2.**
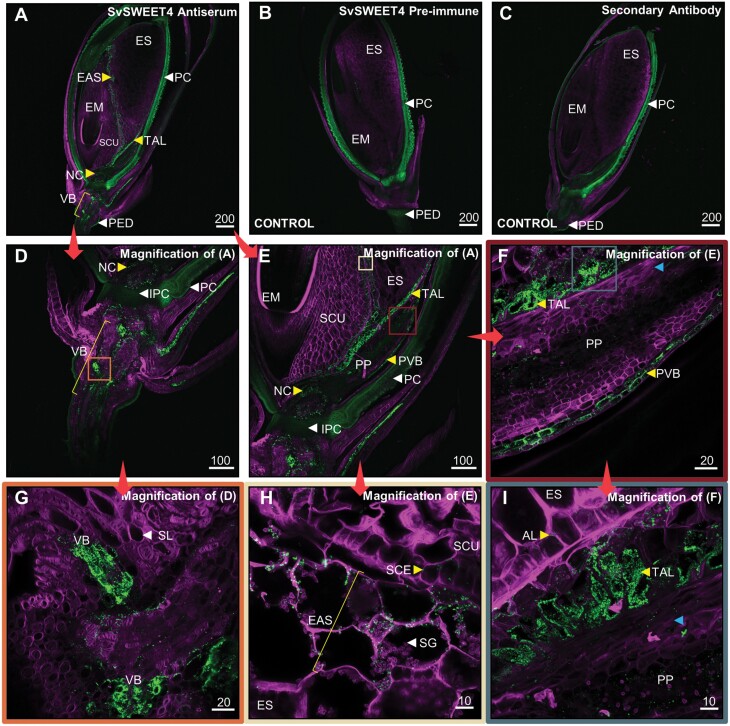
Immunolocalisation of SvSWEET4 within mature *Setaria viridis* (A.10) seed. Immunodetection of SvSWEET4a (A) is contrasted with the pre-immune serum (B) and secondary antibody-only- (C) treated seed sections. Micrographs (D–I) are magnified regions of (A). Arrows and coloured boxes indicate micrographs that are magnified. Autofluorescence of the pedicel (PED), pericarp (PC), and inner pericarp (IPC) is indicated by white arrowheads in (A–E). Immunolabelling (yellow arrowheads) is observed on the vascular bundle (VB) in (D) and (G); the transfer aleurone (TAL) and placental vascular bundle (PVB) in (E), (F), and (I); and the endosperm adjacent to scutellum (EAS) layer in (H). Some weak immunolabelling is observed in the nucellus (NC) in (D) and (E), scutellar epithelium (SCE) in (H), and aleurone (AL) cells in (I). The crushed cells of the placental pad (PP) are indicated by blue arrowheads in (F) and (I). Endosperm (ES); embryo (EM); scutellum (SCU); sclerenchyma (SL); and starch granules (SG). Micrographs are representative of multiple sections. Fluorescence signals are pseudo-coloured: green—protein of interest labelled with secondary antibodies conjugated with Alexa Fluor 488 (excitation, 493 nm; emission, 517 nm); magenta—cell walls (excitation, 254 nm; emission, 432 nm). Scale bars represent µm.

The SvSWEET4 antiserum was subsequently used for immunolocalization on longitudinal Setaria A.10 seed sections. The general structure of the seed consisted of the filial tissue containing the embryo (EM) with the scutellum (SCU) separating it from the endosperm (ES) surrounded by the maternal pericarp (PC), typical of other cereals (Fig. 2A–C) (Esau, 1977). SvSWEET4 showed specific immunolabelling; however, there was autofluorescence observed on the pericarp and pedicel (PED), also apparent in the pre-immune and secondary antibody-only-treated sections (Fig. 2B, C). The commercially available PIP aquaporin antiserum was used as an additional control (Supplementary Fig. S7F, G). No non-specific labelling was observed on the pre-immune and secondary antibody-only-treated sections (Fig. 2B, C; Supplementary Fig. S8B). SvSWEET4 was present in the vascular bundles (VBs) of the pedicel below the inner pericarp (IPC) (Fig. 2A, D, G; Supplementary Fig. S7A–E). Above the IPC, there was weak labelling of SvSWEET4 within the nucellus (NC) (Fig. 2A, E; Supplementary Fig. S7C). The placental pad (PP) structure was named in accordance with its previous identification in *Setaria lutescens*, *Briza maxima*, and *Enneapogon desvauxii*, characterized by crushed cells (Rost and Lersten, 1970; Rost, 1973; Rost *et al*., 1984, 1990) (Fig. 2A, F, I). Adjacent to this structure is what was deemed the placental vascular bundle (PVB), where immunolabelling of SvSWEET4 was also detected (Fig. 2A, E, F). Immunolabelling was observed within the highly invaginated transfer aleurone (TAL) above the PP, with some weak signal on the cuboidal aleurone (AL) cells (Fig. 2F, I). SvSWEET4 was also present in the endosperm adjacent to the scutellum (EAS) layer, which was recently named in maize kernels and found within the endosperm (Doll *et al.*, 2020) (Fig. 2A, E, H; Supplementary Fig. S8A). As characterized previously, the EAS layer contains 2–3 cell layers of the endosperm beside the scutellum. This was the only region labelled with SvSWEET4 antiserum within endosperm. There was also a weak signal present within the scutellar epithelium (SCE).

**Fig. 3.**
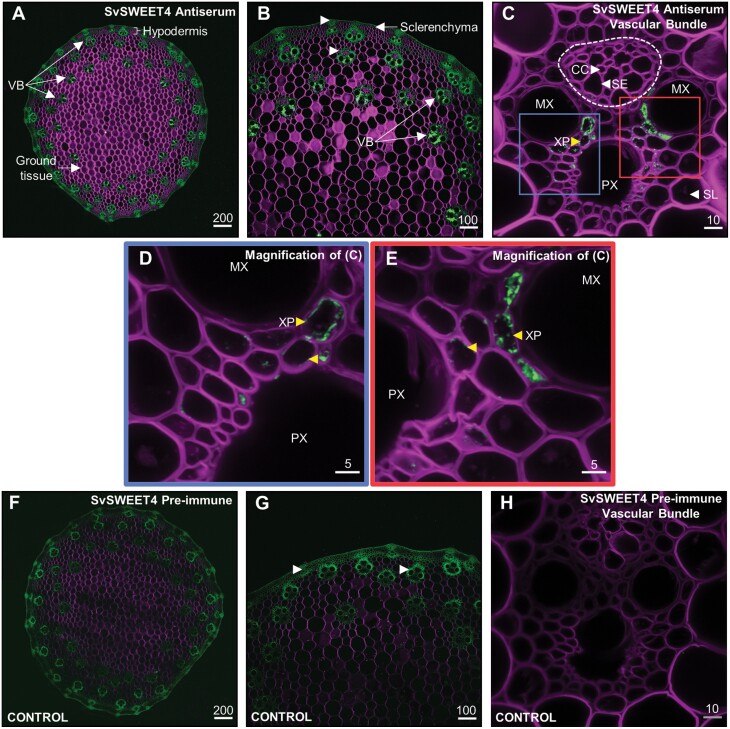
Immunlocalization of SvSWEET4 within developing *Setaria viridis* (A.10) stem. Immunodetection of SvSWEET4 (A–E) is contrasted with the pre-immune serum (F–H) in stem sections. Micrographs (D) and (E) are magnified regions of (C). Autofluorescence of some cells in the vascular bundle (VB) and highly lignified hypodermis is indicated by white arrowheads in (B) and (G). Immunolabelling (yellow arrowheads) is observed in the plasma membrane and possibly the web-like endoplasmic reticulum of xylem parenchyma (XP) adjacent to the protoxylem (PX) and metaxylem (MX) within the VB as indicated by yellow arrowheads in (C–E). The phloem is indicated by the broken line, made up of companion cells (CC) and sieve elements (SE). The sclerenchyma surrounds the VB. Micrographs are representative of multiple sections. Fluorescence signals are pseudo-coloured: green—protein of interest labelled with secondary antibodies conjugated with Alexa Fluor 488 (excitation, 493 nm; emission, 517 nm); magenta—cell walls (excitation, 254 nm; emission, 432 nm). Scale bars represent µm.

Since previous studies have alluded to the presence of SWEET4 within stems (Martin *et al.*, 2016; McGaughey *et al.*, 2016; Mizuno *et al.*, 2016; Hu *et al.*, 2018), immunolocalization was carried out in this tissue from Setaria A.10. The transverse stem section of Setaria consisted of mostly ground parenchyma tissue with VBs in the outer perimeter of the stem, consistent with the general structure of monocot stems (Fig. 3A, B) (Esau, 1977). Specific immunolabelling of SvSWEET4 was observed on the stem sections, although there was autofluorescence of the highly lignified cells of the hypodermis and VBs (Fig. 3A, B, F–H). There was no non-specific labelling with the SvSWEET4 pre-immune serum in the stem (Fig. 3F–H). The VBs showed SvSWEET4 present on the cells of the xylem parenchyma (XP) toward the centre of the VB adjacent to the proto- and metaxylem (PX, MX) (Fig. 3C–E). No labelling was present in the sieve element (SE) or companion cells (CCs) that make up the phloem.

### SvSWEET4a transports glucose and sucrose in Xenopus oocytes

**Fig. 4.**
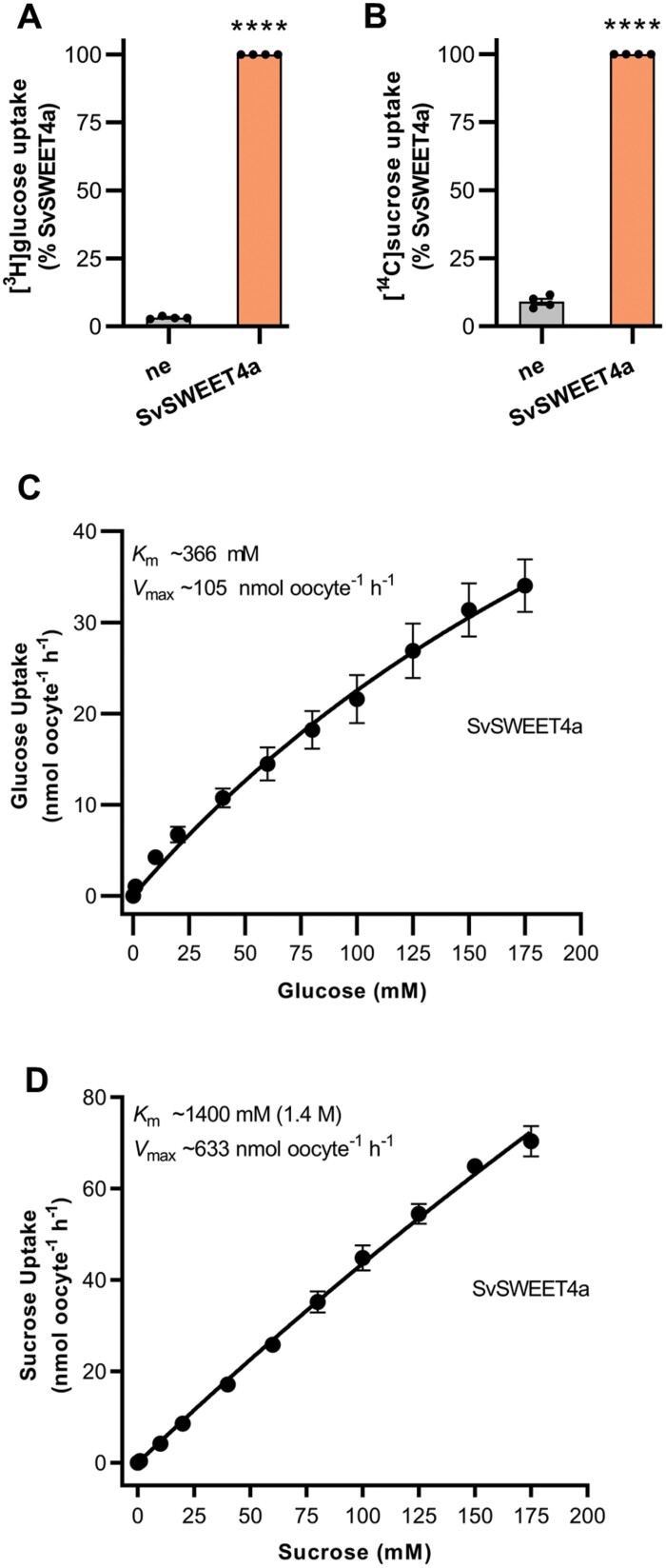
Functional characterization of SvSWEET4a using Xenopus oocytes. The SvSWEET4a-mediated transport of [^3^H]glucose (A) and [^14^C]sucrose (B). The concentration dependence of SvSWEET4a-mediated transport of [^3^H]glucose (C) and [^14^C]sucrose (D). The estimated app *K*_m_ and app *V*_max_ values of sugar transport via SvSWEET4a are indicated in (C) and (D). The data are the mean of at least three independent experiments (each yielding similar results and overlaid as individual data points in A and B) and the error is the SEM. Where not visible, the error bars fall within the symbols. The asterisks denote a significant difference from non-expressing oocytes; ****P<0.0001 (unpaired Student’s *t*-test). Non-expressing oocytes (ne) were used as a negative control.

To examine the sugar substrates and transport kinetics of SvSWEET4a, the protein was expressed in Xenopus oocytes. Glucose and sucrose were investigated as potential substrates since they are the predominant soluble sugars generally present in plant tissues, and are reported to be important sugars in sink tissue of other cereals (Hassid, 1967; Arnold, 1968; Weber *et al.*, 1995; Weschke *et al.*, 2003; Martin *et al.*, 2016; Milne *et al*., 2017a, b, 2018). The accumulation of [^3^H]glucose and [^14^C]sucrose was only ~3% and 9%, respectively, for the negative control non-expressing oocytes relative to oocytes expressing SvSWEET4a (Fig. 4A, B). An analysis of the concentration dependence of sugar transport via SvSWEET4a yielded an apparent *K*_m_ (app *K*_m_) of ~366 mM for glucose with an apparent *V*_max_ (app *V*_max_) of ~105 nmol oocyte^–1^ h^–1^. SvSWEET4a-mediated sucrose transport yielded an app *K*_m_ of ~1.4 M, with the app *V*_max_ being ~633 nmol oocyte^–1^ h^–1^ (Fig. 4C, D). However, it must be noted that saturation of transport of both glucose and sucrose via SvSWEET4a was not reached due to physiological limitations of the oocyte system (exceeding the concentration of sugar in the extracellular solution beyond 175 mM caused excessive osmotic stress for the oocytes).

### Changes in Setaria seed carbohydrate content during development

**Fig. 5.**
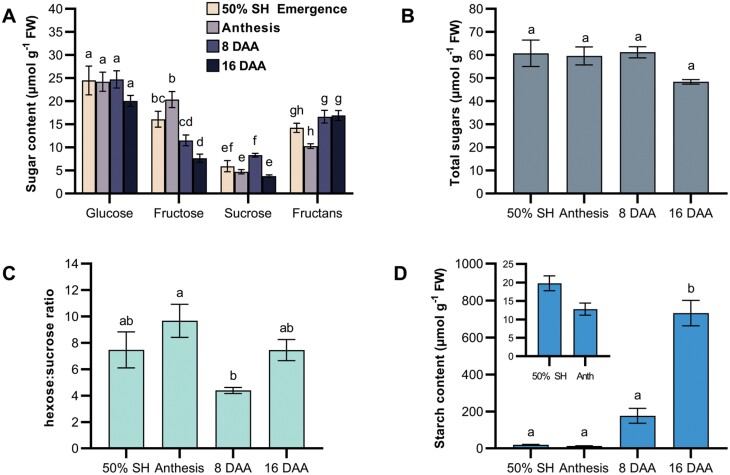
Major carbohydrates of *Setaria viridis* A.10 seed heads at different developmental stages. Plants harvested at 50% seed head emergence (50% SH), anthesis, 8 DAA, and 16 DAA for carbohydrate analysis (A–D). Soluble sugars (A), total sugars (B), hexose:sucrose ratio (C), and starch (D) are displayed. For (D) the inset shows starch values at 50% SH emergence and anthesis that are not distinguishable. Bars represent the mean of four biological replicates, where each replicate was pooled from four plants. Error bars denote SEM. Letters denote significance (adjusted *P*<0.05) between developmental stages for each carbohydrate as determined using a one-way ANOVA with Tukey’s post-hoc test for multiple comparisons.

In the well-studied cereals rice, wheat, barley, and maize, there are defined changes in carbohydrate composition of grains during post-anthesis development as the tissue transitions from cell division and expansion to starch synthesis and grain ‘filling’ (Lim and Gifford, 1984; Krishnan and Dayanandan, 2003; Li *et al.*, 2013). The dominant sugars present in the reproductive structure will have a bearing on the nature and properties of sugar transporters required for apoplastic sugar movement, and these have been correlated with expression levels of sugar transporters in cereal crop grains (Weber *et al*., 1997a, b; Weschke *et al.*, 2003). As these processes have not been characterized in Setaria, sugar and starch content during seed development was quantified in seed heads from four developmental stages: 50% seed head emergence, anthesis, 8 DAA, and 16 DAA. Seed heads were sampled from the widely studied genetic model known as the A.10 ecotype and from the ME034V ecotype (Fig. 5; Supplementary Figs S1, S9), which is more amenable to genetic transformation (Brutnell *et al.*, 2010; Van Eck *et al.*, 2017; Thielen *et al.*, 2020). Setaria seeds and millets in general have many small seeds compared with other cereals such as sorghum, maize, and rice; therefore, harvesting single seeds from an inflorescence was impractical and data shown are from whole seed heads (Fig. 5).

Glucose content remained relatively unchanged (20–25 µmol g^–1^ FW) during seed head development in A.10 with no significant differences between stages (Fig. 5A). Fructose centent increased from 50% seed head emergence to anthesis, where it subsequently halved (significantly) at 8 DAA. Sucrose content fluctuated throughout development between 4 µmol g^–1^ FW and 6 µmol g^–1^ FW in the first two stages harvested and then significantly increased from anthesis to 8 DAA, later decreasing to ~3.8 µmol g^–1^ FW at 16 DAA. Fructans were also present in A.10 seed heads, increasing ~1.6-fold from anthesis to 16 DAA. Total soluble sugars did not change significantly through the course of seed development (Fig. 5B). To examine whether a shift from high hexose to high sucrose was apparent, as has been observed during grain development in other species (Cheng and Chourey, 1999; Weschke *et al.*, 2003), the hexose:sucrose ratio was examined throughout development (Fig. 5C). There was a significant decrease in the hexose:sucrose ratio between anthesis and 8 DAA; however, this increased again at 16 DAA in A.10. From 8 DAA there was a large increase in starch accumulation (~13.8-fold compared with anthesis) (Fig. 5D). Starch content further increased, significantly (~4.1-fold) between 8 and 16 DAA. Although A.10 and ME034V ecotypes mostly exhibited the same carbohydrate content measured, there were some differences in the glucose and sucrose amounts and thus the hexose:sucrose ratio (Supplementary Fig. S9).

### Transcriptional profiling of sugar transporters and enzymes during seed development in Setaria

**Fig. 6.**
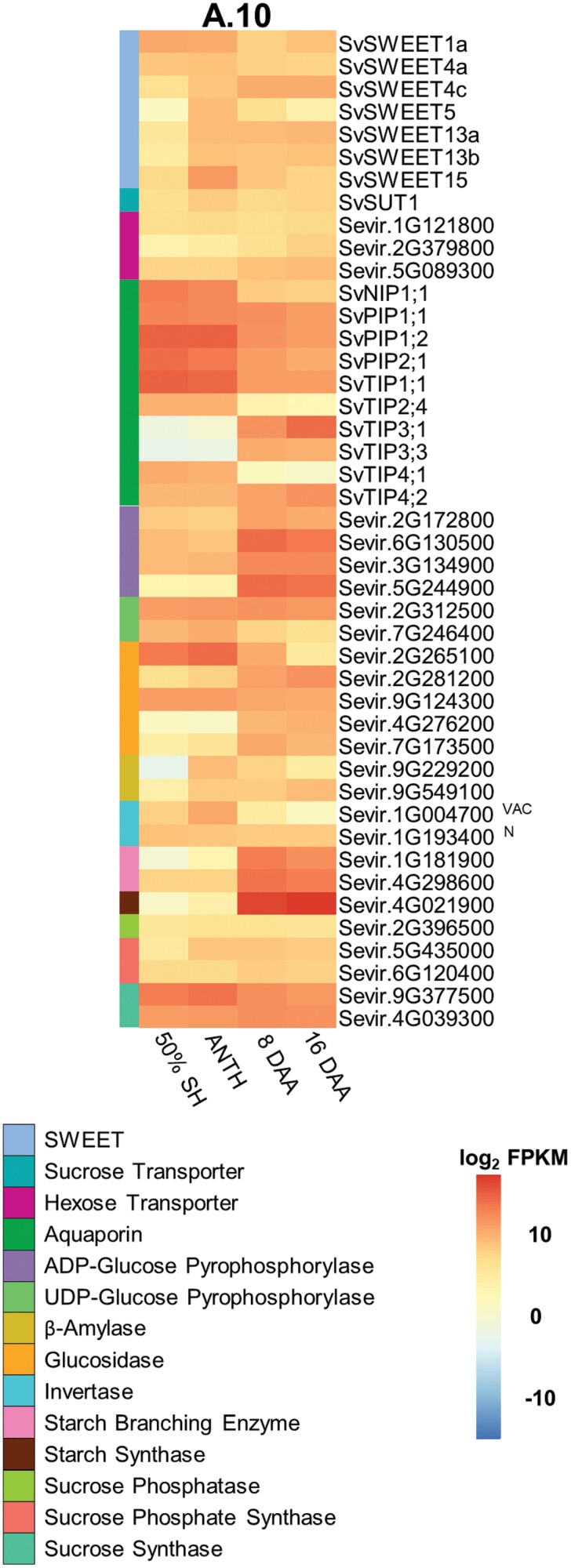
Expression of genes encoding sugar transporters and enzymes involved in sugar metabolism within *Setaria viridis* seed heads. Heatmaps displaying log_2_ FPKM values of select genes encoding proteins involved in sugar transport or metabolism in seed heads of *Setaria viridis* A.10. Whole seed heads were harvested at four stages of development: 50% seed head emergence (50% SH), anthesis (ANTH), 8 DAA, and 16 DAA. Values represent the mean from four biological replicates where each replicate is pooled from four plants. Row colours denote gene families. The different types of invertases are indicated beside their gene IDs: neutral (N), and vacuolar (VAC).

Thus far, SvSWEET4 has been shown to probably have a role in the post-phloem pathway of sink tissues, particularly in the seed. To determine if SvSWEET4 and/or other SWEETs were temporally expressed during seed head development, poly(A)-enriched RNA-sequencing (RNAseq) was carried out (Fig. 6; Supplementary Fig. S10). The expression of *SWEET* genes was compared with that of the genes that encode key proteins involved in carbohydrate metabolism during the seed filling process so that transport, metabolism, and carbohydrate content could be correlated. A comprehensive list of genes encoding enzymes and transporters in seed filling was collated to examine their expression at different stages of development in A.10 and ME034V ecotypes (Supplementary Dataset S2; Supplementary Figs S3, S10). From this, a subset of the most highly expressed genes was identified from the different gene families encoding sugar-related metabolism and transport proteins in the A.10 ecotype (Fig. 6). qPCR of *SvSWEET4a* was also carried out on leaf, stem, and seed head tissue alongside other *SWEET* genes to provide an overall view of the pattern of expression (Supplementary Fig. S11). These *SWEET* genes were chosen based on previous evidence for their expression in the leaf and/or sinks (Emms *et al.*, 2016; Martin *et al.*, 2016; L. Chen *et al.*, 2022).

*SvSWEET1a* was expressed at higher levels during earlier stages of seed head development at 50% seed head emergence and anthesis. Conversely, *SvSWEET4c* was expressed more abundantly at 8 and 16 DAA. *SvSWEET4a*, which is homologous to *SvSWEET4c*, was generally expressed throughout seed head development. Similarly, in maize, the electronic Fluorescent Pictograph (eFP) expression atlas of *ZmSWEET4* homologues revealed similar results with relatively stable expression throughout seed development (Supplementary Fig. S12). Observations of *SvSWEET4a* expression across the leaf, stem, and seed head highlighted its predominance in sinks (Supplementary Fig. S11). *SvSWEET5* and *SvSWEET15* were the most highly expressed of the *SWEET* gene family and were predominantly expressed during anthesis. *SvSWEET13a* and *SvSWEET13b* gene expression increased from anthesis onwards. *SvSUT1* transcript was present across all stages of development as were *HXT* transcripts. Aquaporins, which are predominantly involved in the passive movement of water and other substrates (Patrick *et al.*, 2001), were highly expressed during earlier stages of Setaria seed head development (notably *SvNIP1;1*, *SvTIP2;4*, and *SvTIP4;1*). Putatively tonoplast-localized aquaporin genes *SvTIP3;1* and *SvTIP3;3* were expressed during later stages of development at 8 and 16 DAA. All other aquaporins were expressed at high levels throughout development.

Invertases, involved in the breakdown of sucrose in sink tissues, were expressed at moderate to high levels throughout development. Genes encoding a vacuolar invertase (*Sevir.1G004700*) were abundant at anthesis. Neutral invertase (*Sevir.1G193400*) was expressed throughout development. Expression of SuSy, one of the most common sink-localized, cytosolic sucrolytic enzymes (Weber *et al.*, 1997b), was highly expressed from 8 DAA. Genes encoding proteins involved in sucrose synthesis, UDP-glucose pyrophosphorylase (UDP-glc PPase), sucrose phosphatase (SPP), and sucrose phosphate synthase (SPS), were generally ubiquitously expressed throughout development. The most abundantly expressed genes encoded ADP-glc PPase, starch synthase (SS), and starch branching enzyme (SBE), all of which are involved in starch synthesis, and up-regulated during maturation stages from 8 DAA. Notably, genes encoding the starch degradation enzyme glucosidase was expressed at the earlier stages (*Sevir.2G265100*) or throughout development (*Sevir9G124300*).

## Discussion

Phloem unloading and the capacity of the post-phloem transport pathway for photoassimilates are major determinants of yield in many cereal crops which are harvested for their grains and/or stems (Braun *et al.*, 2014). In many cereals, it has been established that SUT1 is integral to apoplastic phloem unloading in seeds (Weschke *et al.*, 2000; Furbank *et al.*, 2001; Aoki *et al.*, 2004). Thus far, there has been some evidence for the role of SWEETs in seed filling in maize (Sosso *et al.*, 2015) and rice (Sosso *et al.*, 2015; Ma *et al.*, 2017; Yang *et al.*, 2018; Fei *et al.*, 2021; Li *et al.*, 2022). In maize, *zmsweet4c* and *miniature1* mutants have both been shown to exhibit an impaired seed filling phenotype and lack a fully differentiated basal endosperm transfer layer (BETL), the latter of which has a gene knockout that usually encodes a cell wall invertase specific to the endosperm (Cheng *et al.*, 1996; Sosso *et al.*, 2015). Mutational analysis commonly produces developmental disruptions resulting in aberrant tissue structures, confounding the interpretation of the phenotypes observed because the lack of gene expression could have disrupted transport at any stage of development. Thus, genetic studies provide limited developmental stage-specific information on the role of SWEETs in grain development and do not provide the spatial information on the physical location of these proteins within the reproductive structures needed to support a model of sugar movement. The broad developmental expression of ZmSWEET4 orthologues (Supplementary Fig. S12) makes this issue even more important as a knockout could have a multitude of effects during development at any stage from fertilization to grain filling.

In our study, we produced a number of transgenic Setaria RNAi lines targeting SvSWEET4 but were unable to regenerate plants with a visible phenotype or with a detectable reduction in SWEET4 transcript levels (Supplementary Fig. S13). This could reflect a role for this SWEET in the regeneration and tissue culture process or species-specific functional redundancy, which has been highlighted in previous studies (Chen *et al.*, 2015; Bezrutczyk *et al.*, 2018). Nevertheless, the analysis of the CDS of the three SvSWEET4 isoforms highlighted that the high similarity between them would not enable specific regions of each to be targeted for individual gene knockdowns or knockouts (Supplementary Fig. S5E). Investigation of the functions of SWEETs has so far not been expanded outside a small subset of cultivated cereals and represents a large gap in our knowledge considering that inflorescence and seed/grain anatomy can differ drastically across C_4_ grasses unlike in the commonly studied C_4_ crops sorghum and maize (Zee and O’Brien, 1970; Rost, 1973; Rost *et al*., 1984, 1990; Evers and Millar, 2002; Wang *et al.*, 2012). Here, we expand upon those results for the C_4_ monocot *S. viridis*, likely to be pertinent to the anatomically similar grain of *S. italica* and other related millet crops (Rost and Lersten, 1970; Rost, 1973).

### SWEET4 is a high-capacity transporter and localized to the seed and stem tissues

SWEET4 homologues have been implicated in the phloem unloading and post-phloem sugar transport pathway of sink tissues where they are generally highly expressed compared with source tissues (Supplementary Fig. S11) (Sosso *et al.*, 2015; Martin *et al.*, 2016; Mizuno *et al.*, 2016; Hu *et al.*, 2018). The knockout lines *zmsweet4c* and *ossweet4* had previously been shown to have impaired seed filling (Sosso *et al.*, 2015). This work analysed the transport properties and localization of SWEET4 and suggests a role in the pathway of sugar movement in C_4_ grasses.

The antisera detected a strong band at ~20 kDa in lysate of oocytes expressing SvSWEET4 not present in non-expressing oocytes (Fig. 1). As the predicted size of the SvSWEET4 monomer is ~27 kDa, this difference in apparent molecular mass may be attributable to varying post-translational modifications in each system (Niittylä *et al.*, 2007; Q. Chen *et al.*, 2022). Notably, SvSWEET4 proteins were present as dimers in plant extracts despite using denaturing conditions (Fig. 1). It is possible that the oocyte protein translation machinery formed weaker bonds during oligomerization of SvSWEET4 than in plants, and that sample preparation of oocyte lysates is sufficient to break these bonds. Previous studies have shown that the stronger bonds formed in plant-derived oligomers appeared not to be broken by standard denaturing treatments, suggesting that a covalent linkage occurs during extraction or assembly to the membrane (L. Chen *et al.*, 2022). SWEETs are functional as a dimer to form the necessary pore size required to accommodate substrates (Xuan *et al.*, 2013).

Using immunolocalization, SvSWEET4 was detected at the maternal–filial and filial–filial cell interfaces of the Setaria seed. Previously, it has been shown that SUT1 functions during the grain filling process of rice and wheat, and is usually found at the interface between the maternal and filial cells (Bagnall *et al.*, 2000; Furbank *et al.*, 2001). Since SWEETs and SUTs are often co-expressed in the same or adjacent cells (Hu *et al.*, 2021), it is unsurprising that SvSWEET4 was also found at similar interfaces (Fig. 2). Presumably in Setaria, photoassimilates that reach the placental vascular bundle through the symplast must be exported into the apoplast, which we propose is mediated by SvSWEET4 (Fig. 2E, F). The placental pad, where the symplastic barrier between maternal and filial tissues probably occurs in Setaria, and in all species examined thus far, is then the site for the majority of sugar flux out of maternal tissues into filial tissues across the highly invaginated transfer aleurone layer (Pate and Gunning, 1972). Once photoassimilates reach the placental pad cells, they could move freely from cell to cell symplastically and subsequently into the apoplast at the filial interface. The cells within the placental pad are thought to be senescent and have been observed to contain plasmodesmatal pits, probably suggesting there are symplastic connections between these cells (Zee and O’Brien, 1971; Rost, 1973; Rost *et al.*, 1984).

**Fig. 7.**
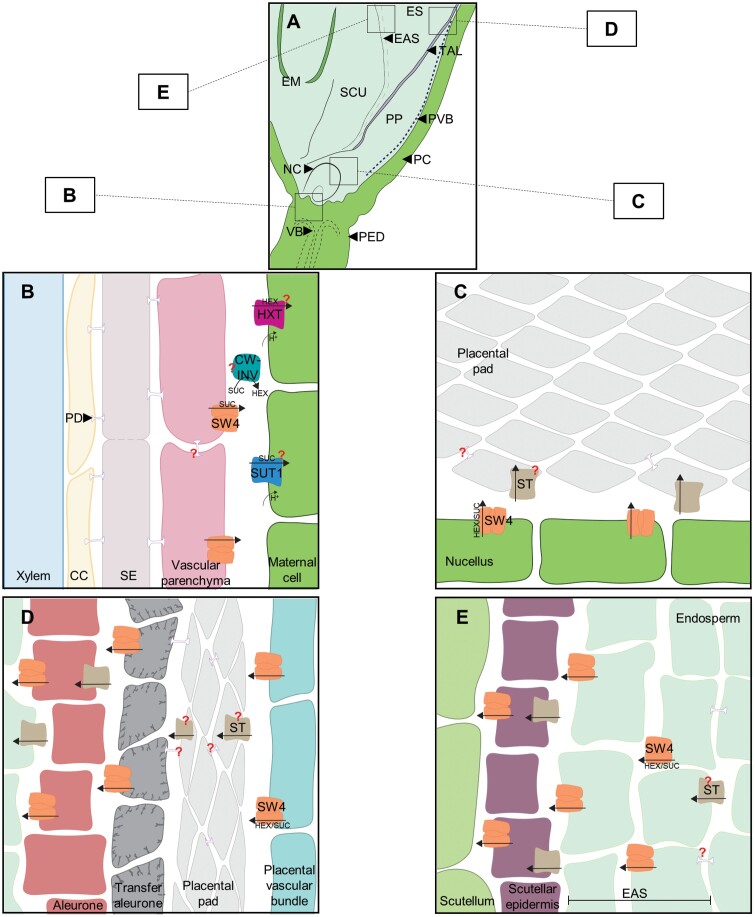
Hypothetical model for the movement of photoassimilates within the Setaria seed. A simplified longitudinal section of the mature Setaria seed (A). Symplastic phloem unloading of sucrose (SUC) via plasmodesmata (PD) from the sieve element–companion cell (SE–CC) complex can be moved into the vascular parenchyma (VP) where SvSWEET4 (SW4; orange) exports it into the apoplast within the vascular bundle (VB) (B). Once in the apoplast, cell wall invertase (CW-INV; teal) may hydrolyse SUC into hexoses (HEX) for import by hexose transporters (HXT; magenta) into other maternal cells (MCs) such as the adjacent sclerenchyma cells or nucellus (NC). Alternatively, if SUC is not broken down, it is imported by SUT1 into the MCs. Photoassimilates that reach the nucellus are exported by SvSWEET4 and could be imported by a sugar transporter (e.g. HXT, SUT, or possibly a SWEET) (ST; beige) into the placental pad (PP) (C). Sugars that have reached the placental vascular bundle (PVB) are exported by SvSWEET4 into the apoplast and possibly taken up into the adjacent PP cells by an ST (D). Sugars move either sympastically or apoplastically into the highly invaginated transfer aleurone cells (TAL), exported by SvSWEET4, and then imported into the cuboidal aleurone cells (AL) to be again exported by SvSWEET4. As sugars cross the endosperm (ES) they reach the endosperm adjacent to scutellum (EAS) layer, exported by SvSWEET4, and imported into the scutellar epithelium (SCE) lining the scutellum (SCU) and ES interface where they are exported by SvSWEET4 again (E). PD connections between filial and maternal cells are not well understood in cereal seeds. Pedicel (PED); pericarp (PC).

Highly invaginated transfer cells, indicative of a region where a high level of nutrient flux must occur, are almost always seen in the filial tissues at the maternal–filial interface, and these are present in Setaria seeds (Fig. 2E, F, I; Supplementary Fig. S8A). SWEET4 protein abundantly decorated the membranes of these cells, suggestive of a role in high-capacity transport of glucose and/or sucrose at this interface to support the growing embryo and starchy endosperm of cereals (Rost and Lersten, 1970; Zee and O’Brien, 1971; Pate and Gunning, 1972; Maness and McBee, 1986). It was not possible to determine any ‘sidedness’ of expression which would help to determine whether SWEET4 was importing sugar into these cells down a gradient from the placental pad apoplast or facilitating transport between transfer cells and the underlying filial cell layers (Fig. 7D). In species such as wheat, barley, and rice, uptake of sucrose from the apoplast by the aleurone/modified aleurone layer is catalysed by the active transporter SUT1 during grain filling (Weschke *et al.*, 2000; Furbank *et al.*, 2001; Aoki *et al.*, 2004), suggesting that the latter option may be the case. Since SWEET4 can transport both glucose and sucrose, cell-specific sugar composition and sugar gradient measurements would be useful in delineating the mechanism of post-phloem sugar transport in this seed. Unfortunately, literature is scarce on carbohydrates measured within specific structures of the seed. Where there is a marked endosperm cavity, such as wheat, careful and laborious use of aphid stylet exudates (Fisher and Gifford, 1986) suggested that the major sugar in this tissue is sucrose and there is a 10-fold gradient from the phloem sieve tubes to the endosperm cavity. However, in the placental sac of sorghum, it was shown that hexoses would reach up to 40 g l^–1^ in different varieties of sorghum with <10 g l^–1^ of sucrose (Maness and McBee, 1986). These sugar amounts equate to sugar concentrations of ~220 mM for glucose and 30 mM for sucrose; *K*_m_ values obtained here for SWEET4 were 366 mM for glucose and 1.4 M for sucrose, considerably higher than the sugar content reported in the sorghum placental sac. Higher amounts of hexoses within the placental pad, well below the *K*_m_ of SWEET4 for glucose, and the affinity for sucrose relative to measured amount might suggest that SvSWEET4a predominantly exports hexoses at the transfer aleurone interface for it to reach the endosperm.

SvSWEET4 protein was also present at high levels in the EAS layer; tissue recently named in maize (Fig. 2A, E, H; Supplementary Fig. S8A) (Doll *et al.*, 2020). Previous transcriptional studies on maize have implicated SWEETs at the EAS layer and BETL (Sosso *et al.*, 2015; Doll *et al.*, 2020). While these studies only presented transcriptional data on SWEETs, immunodetection of SvSWEET4 protein carried out on equivalent structures in the Setaria seed confirm a role in these tissues. The presence of high-capacity SWEETs at the TAL and EAS is consistent with a physiological role in transporting large amounts of sugars into the endosperm down a concentration gradient during seed filling to support the high flux of sugars necessary for starch synthesis and embryo development (Figs 4C, D, 5). Moreover, it is interesting to note that the estimated *K*_m_ and *V*_max_ values of SvSWEET4a are much higher than previously published values (Chen *et al*., 2010, 2012; Chong *et al.*, 2014; Breia *et al.*, 2020; L. Chen *et al.*, 2022). A high transport capacity was also observed from SWEETs found in the source leaf of Setaria (L. Chen *et al.*, 2022). This might collectively indicate that SWEETs from C_4_ cereals can accommodate higher fluxes compared with C_3_ orthologues. Alternatively, SWEETs that predominate in sinks can reach high transport capacities to support growth and development, at least for SvSWEET4a (Fig. 4C, D). These results provide a foundation for further exploration of how the localization of SWEETs is linked with biochemistry. Nonetheless, given the promiscuity of some SWEETs, SvSWEET4a could equally transport sucrose down a sugar gradient generated by invertase activity. Such activity would subsequently build up a hexose gradient for ongoing transport of reduced sugars via SvSWEET4a to adjacent cells for rapid metabolic consumption (Fig. 7). This could be confirmed by immunolocalization of specific invertase isoforms to individual cell types within the seed structure at different stages of development.

### A role for SvSWEET4 in the vasculature of Setaria sinks

SvSWEET4 protein was also present in maternal tissues along the vascular transport pathway such as the terminal vascular bundles of the pedicel (Figs 2, 3). This suggests that SWEET4 participates early in the phloem unloading pathway or in the immediate steps in the post-phloem pathway (Figs 2A, D, G; 3A; Supplementary Fig. S7). These cells in the vasculature are likely to be parenchyma cells, as was observed in the stem, where localization appeared to be more specifically at the xylem parenchyma (Fig. 3C–E). Prior to the discovery of SWEETs, it was shown in the maize pedicel tissue that photoassimilates were exported from the cells into the apoplast but it was unknown how this occurred (Porter *et al.*, 1985). Solutes might move symplastically down a concentration gradient into parenchyma cells and subsequently be unloaded into the apoplast by SvSWEET4 (Fig. 7B). This would be consistent with the high *K*_m_ of SvSWEET4a for sucrose reported here, which is of the same order as the sucrose concentration present in the phloem (~1 M) (Winter *et al.*, 1992). Sugars can then be utilized for storage elsewhere or used in synthesis of structural carbohydrates, for example in sclerenchyma cells immediately adjacent to the site of unloading (Figs 2G, 3A–E; Supplementary Fig. S7A–E). Interestingly, the localization pattern of SvSWEET4 in the xylem parenchyma was atypical of localization to the plasma membrane and possibly more reminiscent of detection in the endoplasmic reticulum (ER) or Golgi body. These organelles are essential for facilitating the transfer of sugars to proteins, lipids, proteoglycans, and polysaccharides of the plant cell wall (Niemann and Werner, 2015). Consequently, SvSWEET4 could aid in this process given it was found in highly lignified tissue structures of the stem and pedicel (Figs 2, 3). In legumes, xylem parenchyma transfer cells have been observed within the stem, so there is a possibility that SvSWEET4 is located on highly invaginated transfer cells of the stem and pedicel where large fluxes of solutes must occur (Kuo *et al.*, 1980). In Arabidopsis, AtSWEET9 has previously been shown to localize to the plasma membrane, the *trans*-Golgi apparatus, and multivesicular bodies, using green fluorescent protein (GFP)–SWEET fusions (Lin *et al.*, 2014). SvSWEET4 could load vesicles with sugars for exo- or endocytosis or export sugars from the vesicles depending on the sugar gradient. In the rice leaf blade, vesicles have been observed within the xylem parenchyma cells where endocytosis might occur for xylem sap retrieval from the apoplast (Botha *et al.*, 2008). In plants it has been postulated that photoassimilate uptake can also be mediated by endocytosis and is perhaps sucrose induced, although this has not yet been shown *in planta* (Etxeberria *et al*., 2005a, b; Baroja-Fernandez *et al.*, 2006; Aoki *et al.*, 2012; van Bel, 2021). The immunodetection pattern of SvSWEET4 in the xylem parenchyma of stems might provide the first line of evidence for this within the plant.

### Carbohydrate and transcriptional changes of Setaria seed heads

Changes in the glucose:sucrose ratio are important for the development of both dicot and monocot seeds (Weschke *et al*., 2000, 2003; Tiessen and Padilla-Chacon, 2013). In many grasses, glucose is proposed to be produced in the apoplast from the action of cell wall invertase on sucrose leaving the phloem, and this glucose is then imported into the dividing cells of the endosperm by HUTs (Cheng *et al.*, 1996; Cheng and Chourey, 1999; Weschke *et al.*, 2003). After cell division is complete, SuSy is highly expressed in the endosperm tissue, and sucrose itself is transported by SUTs across the aleurone and subaleurone layers of the endosperm to fuel starch synthesis. Thus, by comparing temporal expression patterns of genes encoding enzymes in carbohydrate metabolism with those for sugar transporters, insights into the post-phloem transport process can be derived (Figs 5, 6). In the A.10 ecotype of Setaria studied here, there was no clear transition in sugar composition observed across the developmental stages examined, although there was a significant decrease in the hexose:sucrose ratio from anthesis to 8 DAA, but this increased again at 16 DAA as total soluble sugars decreased, and starch content increased (Fig. 5C, D). However, the ratio was still >1, indicating that there were still higher amounts of glucose at 8 and 16 DAA, and consequently developmental shifts were not a result of the perception of changes in sucrose level (Fig. 5B). This suggests that the developmental shift to starch accumulation post-anthesis in Setaria may not be a direct response to sugar or hormonal signals as in other reports and that a marked shift from hexose to sucrose transport across filial tissues may not occur. These results were similarly mirrored in the ME034V ecotype except there was a significant decrease in glucose from anthesis to 8 DAA. This ecotype was included in this study as focus has shifted in recent literature to this variety in genetic transformation studies (Van Eck *et al.*, 2017; Thielen *et al.*, 2020). Sampling across more stages of development and beyond 16 DAA might serve to clarify this difference as the duration of seed development may differ between ecotypes. Moreover, the sampling of whole seed heads instead of seeds (necessary given the small seed size) may dampen hexose:sucrose shifts and relative transcript abundance. *SWEET*, *HXT*, and *SUT* genes were predominantly stable in their expression levels during seed development, despite the appearance of transcripts encoding metabolic enzymes involved in carbohydrate synthesis increasing with developmental age as expected (Figs 5, 6; Supplementary Figs S9, S10). The transcript abundance of *AQP* genes is consistent with the large flux of photoassimilates and hence water that would be required for seed filling (Figs 5, 6). Interestingly, *SPP* and *SPS* genes encoding key proteins required for sucrose synthesis were only moderately expressed throughout development (Fig. 6). This is consistent with lower amounts of sucrose observed compared with hexoses (Fig. 5).

*SuSy* was constantly expressed at high levels, and a peak in expression was not evident coincident with the breakdown of sucrose within filial cells to fuel starch biosynthesis. Genes of cell wall invertase were expressed at moderate levels throughout development (Supplementary Fig. S10). Apart from sampling effects, the stable expression of these genes could also be related to different post-translational regulation compared with cereals such as maize where these changes are more marked. Transcripts encoding a vacuolar invertase (*Sevir.1G004700*) were also detected at relatively high levels, presumably catalysing sucrose breakdown for temporary vacuolar storage (Fig. 6). This gene might also be a sucrose:(sucrose/fructan) 6-fructosyltransferase (SST or SFT), which is involved in the transferal of a fructosyl group from sucrose to various acceptors in fructan synthesis (Wagner *et al.*, 1983). The nucleotide sequence similarity between invertases and SST/SFTs makes it difficult to distinguish the two. Phyre2 homology-based protein modelling (Kelley *et al.*, 2015) suggested that the Setaria sequence probably encoded an SST or SFT. This would correlate with the unexpected accumulation of fructans within the seed head at all stages sampled (Fig. 5A). The accumulation of fructans in Setaria might be physiologically relevant for maintaining a concentration gradient of sucrose between the phloem and sink tissue while minimizing the accumulation of osmotically active solutes for development and starch accumulation (Fig. 5D) (Peukert *et al.*, 2014).

Collectively, these results from Setaria are not consistent with the maize model of seed filling. SuSy activity in maize increases during seed maturation as rates of carbohydrate accumulation peak (Chourey and Nelson, 1976). This was not apparent in Setaria where *SuSy* transcripts remained relatively high and unchanged throughout development (Fig. 6). In maize, mutants with defective SuSy and cell wall invertase were shown to have a shrunken seed phenotype (Chourey and Nelson, 1976; Chourey, 1981; Miller and Chourey, 1992; Chourey *et al.*, 1998). Cell wall invertase has been thought to be important for mitotic cell division whilst SuSy breaks down sucrose to provide the substrates necessary for starch synthesis. In these mutational studies, the possibility that both these enzymes are important at all stages of seed development cannot be ruled out as the proteins are absent from all tissue types in the seed of these mutants from germination to desiccation and the phenotype in both cases is shrunken, unfilled seeds. In rice and barley, both C_3_ grasses, cell wall invertase is also expressed early in seed development and proposed to be important for establishing a high hexose:sucrose ratio, promoting cell division (Weber *et al.*, 1995; Weschke *et al.*, 2003). Setaria seed did not exhibit these correlations between sugar species, sucrolytic enzyme levels, and patterns of development, which might indicate that this sugar signal is not integral to the control of seed development in this plant species.

SWEETs play a role in transmembrane sugar movement within sink tissue of Setaria, specifically in the post-phloem transport of sugars in the seed. A model is proposed for their function in seeds in Fig. 7. The relatively stable hexose:sucrose ratio throughout grain development, and the unremarkable changes in developmental expression of genes encoding SWEETs, HXTs, or SUTs together suggest that the post-phloem pathway is uncharacteristic of other cereals. The predominant expression of SWEET4 in sinks rather than source tissue of Setaria (see L. Chen *et al.*, 2022) supports a role for this transporter in these pathways of monocots. Localization of SvSWEET4 proteins to a range of symplastically isolated filial cells, the vasculature of the seed, and xylem parenchyma of the stem supports this hypothesis. Moreover, the demonstration that SvSWEET4a is an exceptionally high-capacity, low-affinity transporter of glucose and sucrose is consistent with the high sugar fluxes required to support accumulation of carbohydrate in seed and stem tissues of cereals. These findings presented in *S. viridis* on SWEET4 highlight the need to expand research into cereal crops beyond maize and rice to better understand the post-phloem pathway influencing sink capacity and yield.

## Supplementary data

The following supplementary data are available at *JXB* online.

Fig. S1. Developmental stages of *Setaria viridis*.

Fig. S2. Optimization of the expression and functional assay conditions for the characterization of SvSWEET4a in Xenopus oocytes.

Fig. S3. 3D multidimensional analysis of *Setaria viridis* seed head gene expression profiles.

Fig. S4. Protein alignments of SvSWEETs against SvSWEET4a epitope regions.

Fig. S5. Phylogeny and alignments of SWEET4 homologues.

Fig. S6. Uncropped image of validation of SvSWEET4 antiserum using immunoblotting.

Fig. S7. Immunolocalization of SvSWEET4 to the vascular bundles of mature *Setaria viridis* (A.10) seeds.

Fig. S8. Immunolocalization of SvSWEET4 on transverse *Setaria viridis* (A.10) seeds.

Fig. S9. Major carbohydrates of *Setaria viridis* (ME034V) seed heads at different developmental stages.

Fig. S10. Expression of genes encoding sugar transporters, aquaporins, and enzymes involved in sugar metabolism within *Setaria viridis* seed heads.

Fig. S11. Relative expression of a subset of *Setaria viridis* SWEETs using qPCR.

Fig. S12. Electronic Fluorescent Pictograph (eFP) of *ZmSWEET4* orthologues.

Fig. S13. Expression analysis of *SvSWEET4a* targeted by RNAi in T_2_*Setaria viridis* plants.

Table S1. List of primers used for qPCR of reference genes and SvSWEETs.

Dataset S1. Summary of RNAseq seed head transcriptome read coverage (related to Fig. 6).

Dataset S2. Log_2_ FPKM values (related to Fig. 6).

erad076_suppl_Supplementary_Table_S1_and_Figures

erad076_suppl_Supplementary_Datasets

## Data Availability

All codes used for analyses are available at GitHub (https://github.com/dtrain16/NGS-scripts). All sequencing data generated for this study are accessible at the NCBI’s Gene Expression Omnibus (Edgar *et al.*, 2002) through GEO Series accession number GSE131540 (https://www.ncbi.nlm.nih.gov/geo/query/acc.cgi?acc=GSE131540). A summary of read coverage can be found in Supplementary Dataset S1.

## Abbreviations

DAA: days after anthesis
SUT: sucrose transporter
SWEET: Sugars Will Eventually be Exported Transporter
