# Supplementary material for: The role of SWEET4 proteins in the post-phloem sugar transport pathway of Setaria viridis sink tissues
Source: J Exp Bot. 2023 Mar 8;74(10):2968–86. doi: 10.1093/jxb/erad076 (PMC10560085; doi:10.1093/jxb/erad076)
Supplement: erad076_suppl_Supplementary_Table_S1_and_Figures [file erad076_suppl_supplementary_table_s1_and_figures.pdf]

Supplementary Files

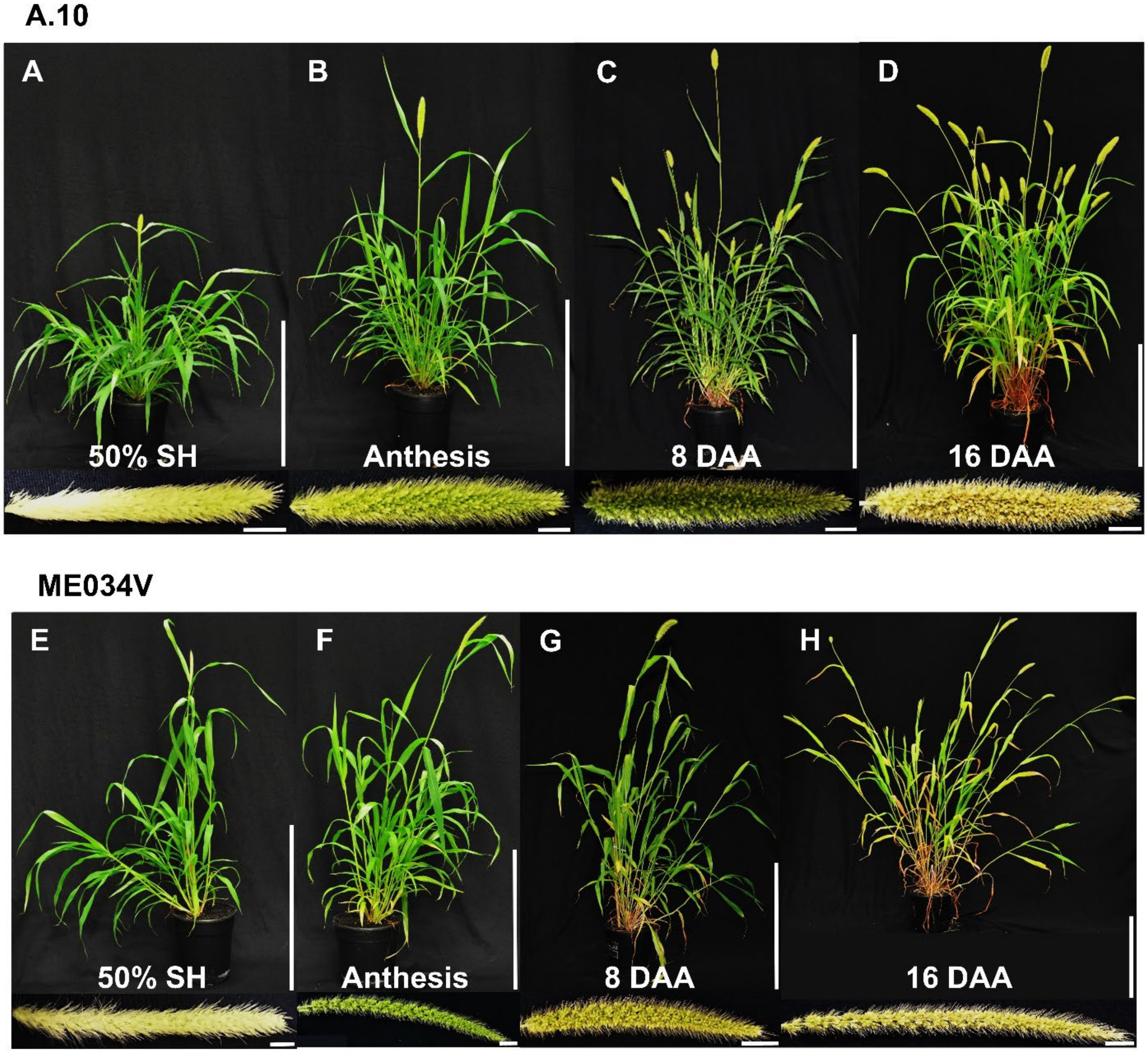

Fig. S1 Developmental stages of *Setaria viridis*.

Whole seed heads were harvested from different developmental stages of A.10 (A-D) and ME034V (E-H) ecotypes. Four stages included: 50% seed head (50% SH) emergence (A,E); anthesis (B,F); 8 days after anthesis (DAA) (C,G); and 16 DAA (D,H). Scale bars represent 30 cm for the whole plant and 1 cm for the seed head in A-H.

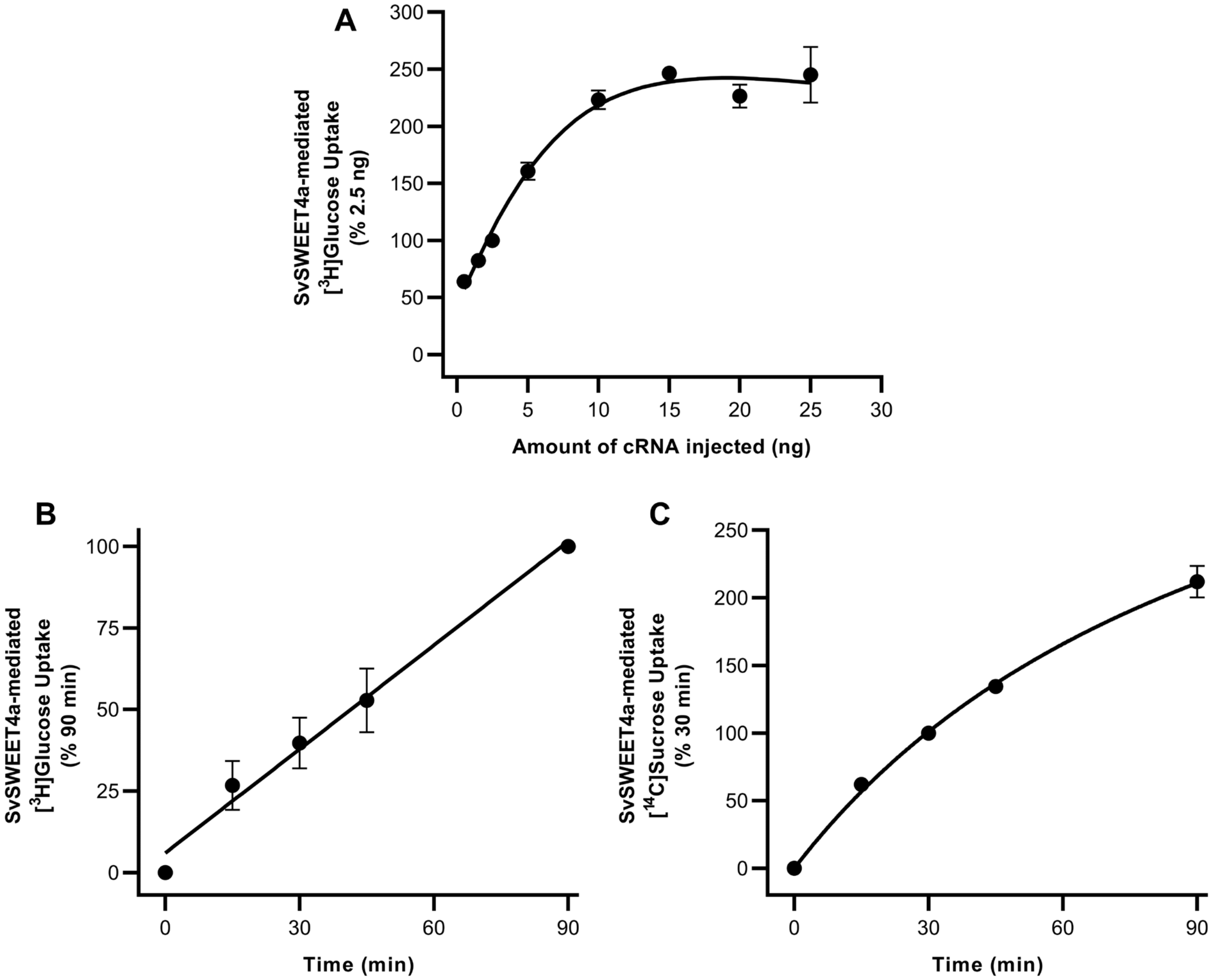

Fig. S2 Optimisation of the expression and functional assay conditions for the characterisation of SvSWEET4a in Xenopus oocytes.

The relationship between the quantity of SvSWET4a cRNA microinjected into the oocyte and the level of SvSWEET-mediated sucrose transport measured indicated that 15 ng was the highest concentration of cRNA within the linear phase (A). Thus, 15 ng of cRNA was used in all subsequent experiments. The transport of [^{3}H]glucose via SvSWEET4a (B) is approximately linear with time until 90 minutes. The transport of [^{14}C]sucrose via SvSWEET4a (C) is approximately linear with time for at least 30 minutes. Uptake concentrations used were a total of 1 mM for glucose and sucrose,

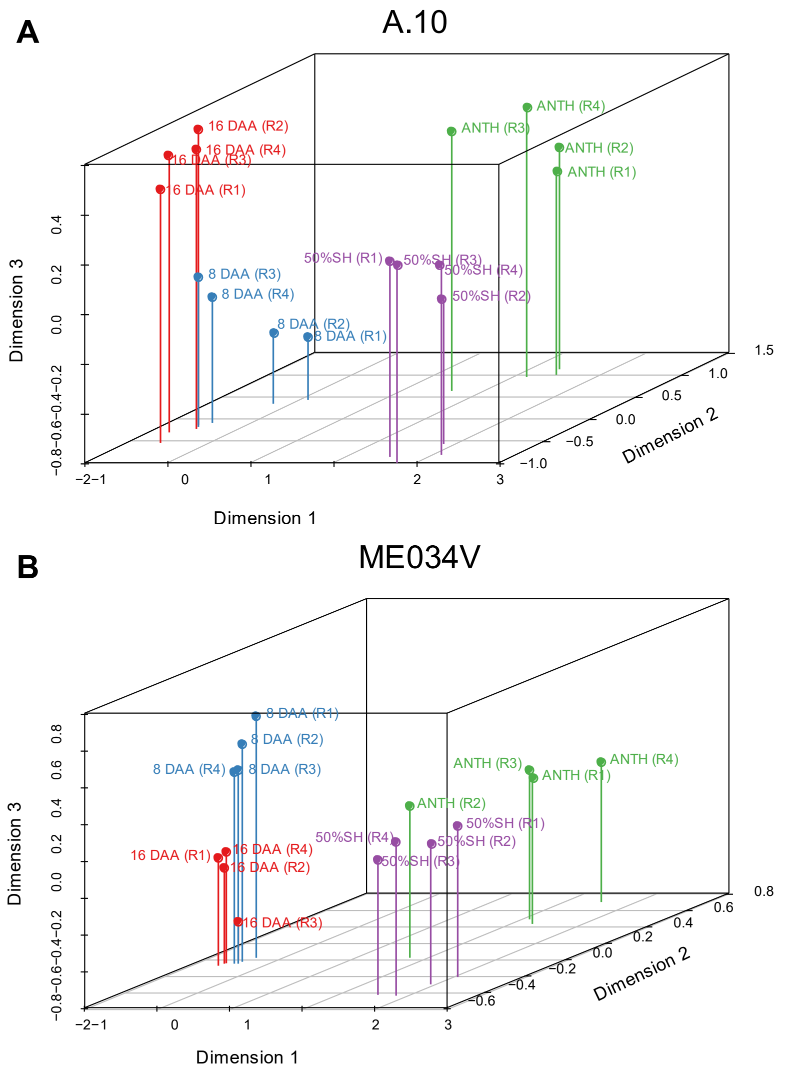

Fig. S3 3D multidimensional analysis of *Setaria viridis* seed head gene expression profiles.

Multidimensional scaling plots of samples harvested at 50% seed head emergence (50% SH; purple), anthesis (ANTH; green), 8 days after anthesis (DAA; blue) and 16 DAA (red) from ecotypes A.10 (A) and ME034V (B). Plot represents the distance between gene expression profiles, based on the biological coefficient of variation calculated on the 500 genes with largest biological variation.

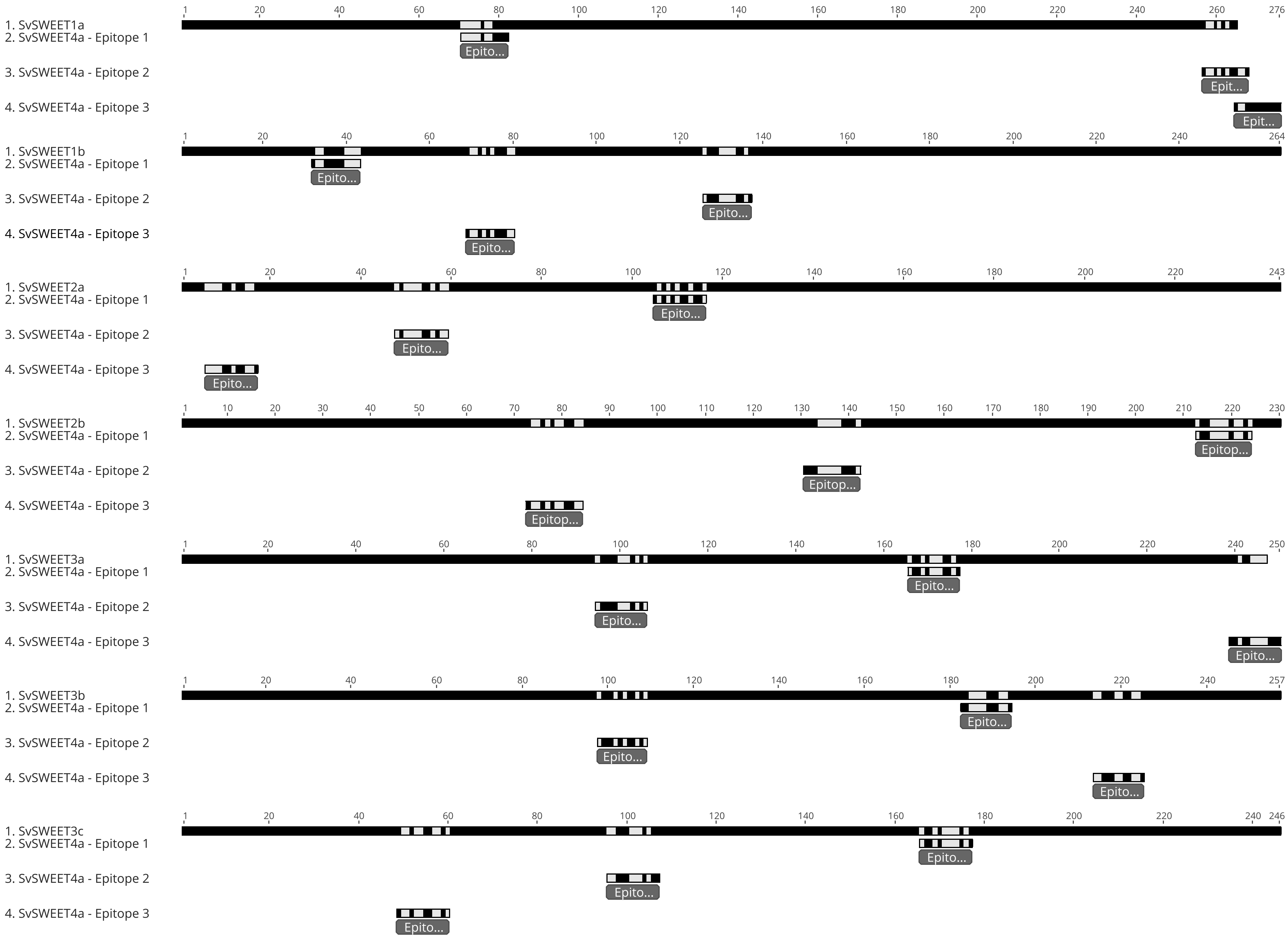
1. SvSWEET1a
2. SvSWEET4a - Epitope 1
3. SvSWEET4a - Epitope 2
4. SvSWEET4a - Epitope 3
1. SvSWEET1b
2. SvSWEET4a - Epitope 1
3. SvSWEET4a - Epitope 2
4. SvSWEET4a - Epitope 3
1. SvSWEET2a
2. SvSWEET4a - Epitope 1
3. SvSWEET4a - Epitope 2
4. SvSWEET4a - Epitope 3
1. SvSWEET2b
2. SvSWEET4a - Epitope 1
3. SvSWEET4a - Epitope 2
4. SvSWEET4a - Epitope 3
1. SvSWEET3a
2. SvSWEET4a - Epitope 1
3. SvSWEET4a - Epitope 2
4. SvSWEET4a - Epitope 3
1. SvSWEET3b
2. SvSWEET4a - Epitope 1
3. SvSWEET4a - Epitope 2
4. SvSWEET4a - Epitope 3
1. SvSWEET3c
2. SvSWEET4a - Epitope 1
3. SvSWEET4a - Epitope 2
4. SvSWEET4a - Epitope 3

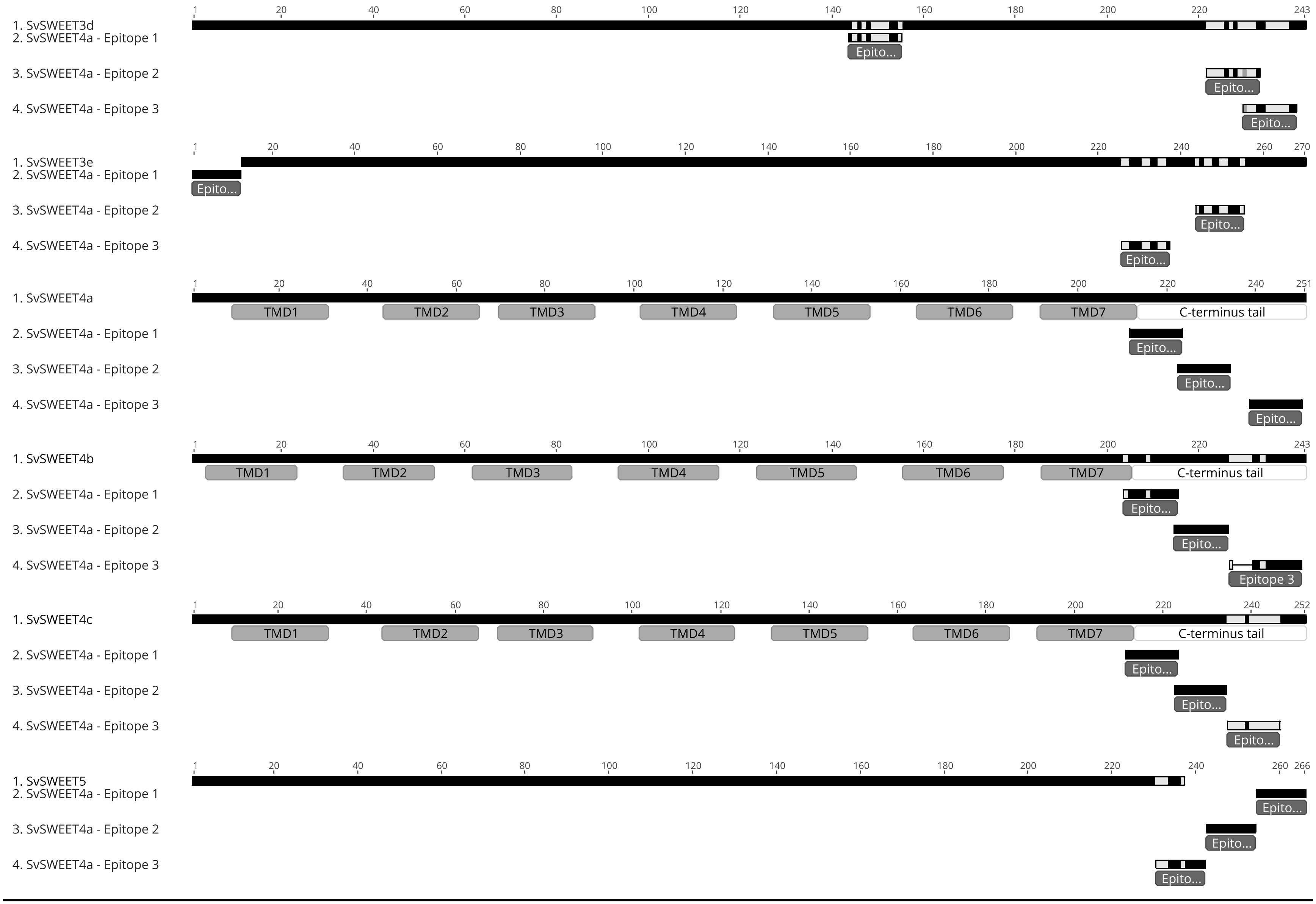
1. SvSWEET3d
2. SvSWEET4a - Epitope 1
3. SvSWEET4a - Epitope 2
4. SvSWEET4a - Epitope 3
1. SvSWEET3e
1. SvSWEET4a
1. SvSWEET4b
1. SvSWEET4c
1. SvSWEET5
TMD1
TMD2
TMD3
TMD4
TMD5
TMD6
TMD7
C-terminus tail
Epito...
Epitope 3

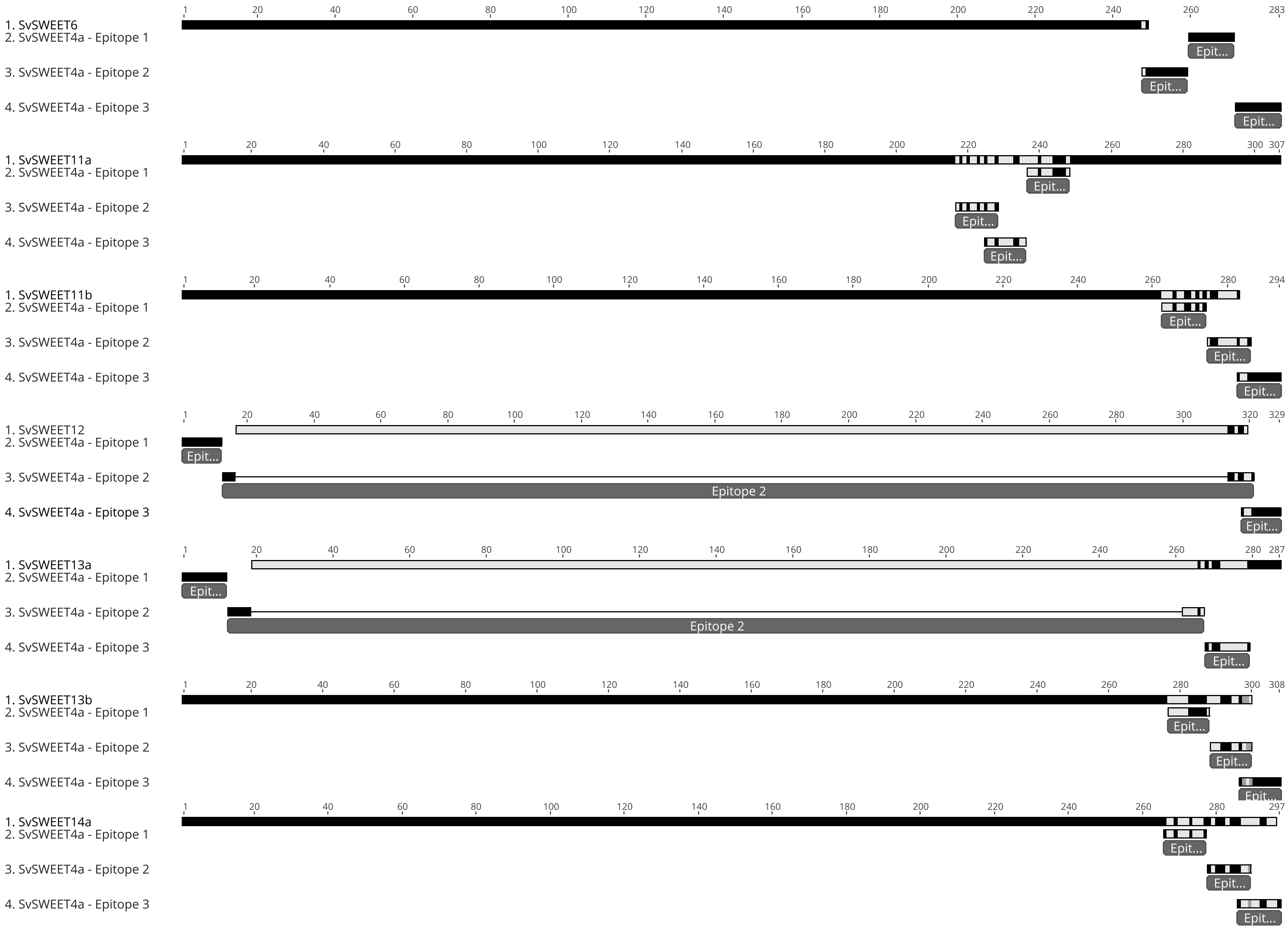
1. SvSWEET6
2. SvSWEET4a - Epitope 1
3. SvSWEET4a - Epitope 2
4. SvSWEET4a - Epitope 3
1. SvSWEET11a
2. SvSWEET4a - Epitope 1
3. SvSWEET4a - Epitope 2
4. SvSWEET4a - Epitope 3
1. SvSWEET11b
2. SvSWEET4a - Epitope 1
3. SvSWEET4a - Epitope 2
4. SvSWEET4a - Epitope 3
1. SvSWEET12
2. SvSWEET4a - Epitope 1
3. SvSWEET4a - Epitope 2
Epitope 2
4. SvSWEET4a - Epitope 3
1. SvSWEET13a
2. SvSWEET4a - Epitope 1
3. SvSWEET4a - Epitope 2
Epitope 2
4. SvSWEET4a - Epitope 3
1. SvSWEET13b
2. SvSWEET4a - Epitope 1
3. SvSWEET4a - Epitope 2
4. SvSWEET4a - Epitope 3
1. SvSWEET14a
2. SvSWEET4a - Epitope 1
3. SvSWEET4a - Epitope 2
4. SvSWEET4a - Epitope 3
Epit...

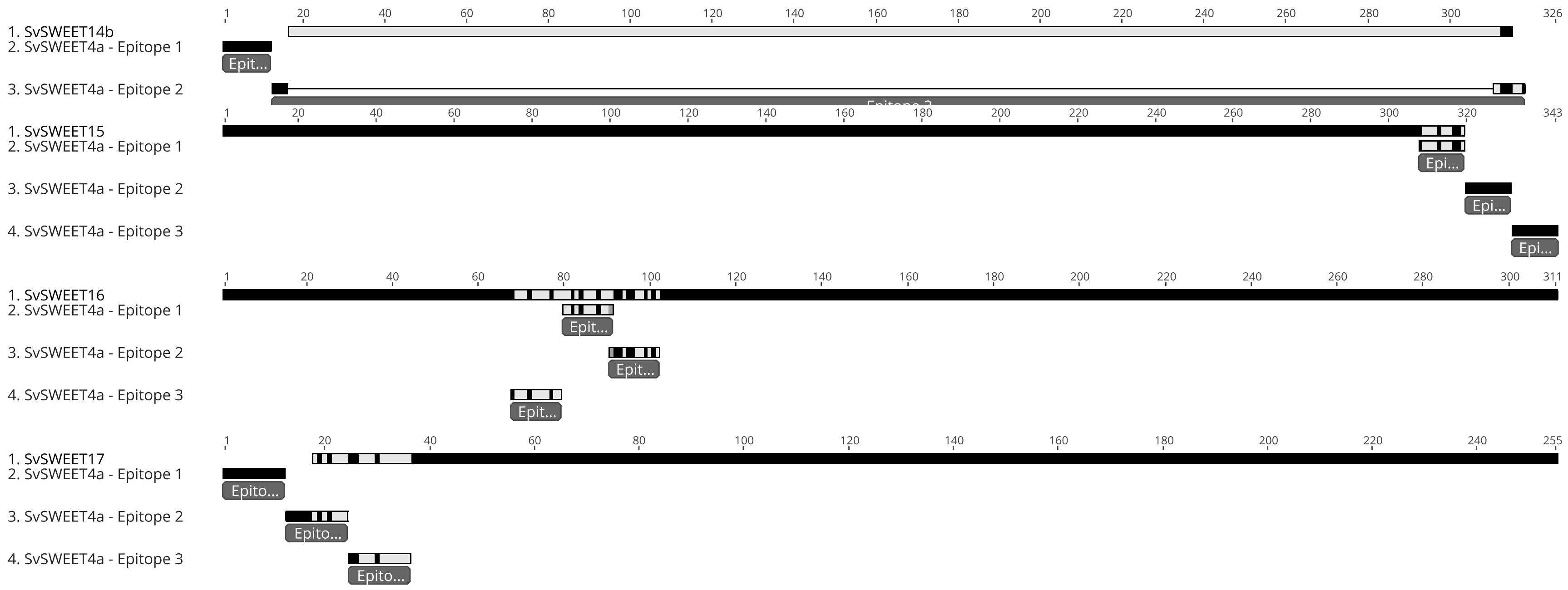

Fig. S4 Protein alignments of SvSWEETs against SvSWEET4a epitope regions.

Three epitope regions were chosen for optimal antigenicity within the C-terminus tail of SvSWEET4a. These regions were aligned with other SWEETs from *Setaria viridis*.

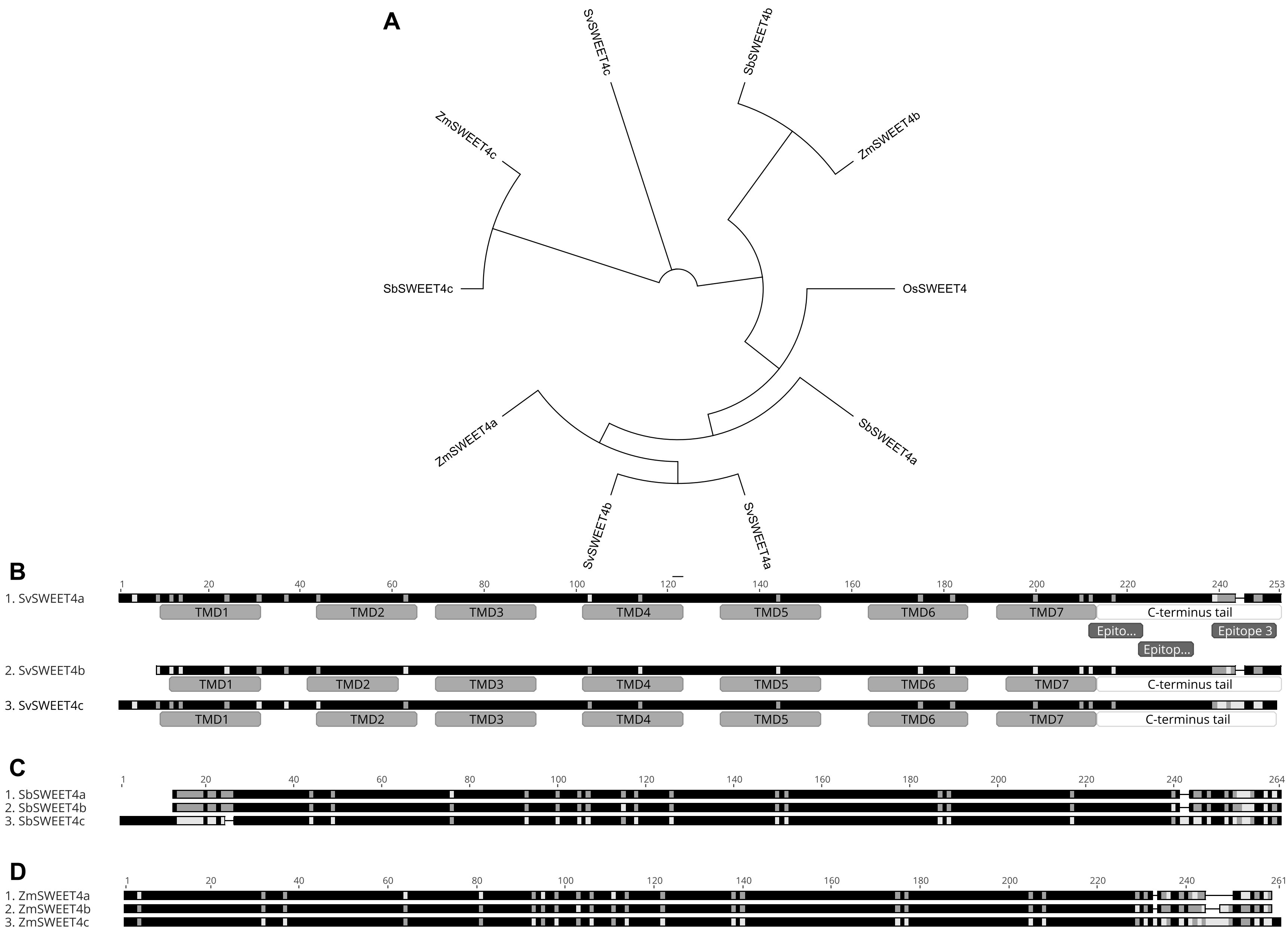
A
SvSWEET4c
SbSWEET4b
ZmSWEET4b
ZmSWEET4c
SbSWEET4c
OsSWEET4
SbSWEET4a
ZmSWEET4a
SvSWEET4b
SvSWEET4a
B
1. SvSWEET4a
2. SvSWEET4b
3. SvSWEET4c
TMD1
TMD2
TMD3
TMD4
TMD5
TMD6
TMD7
C-terminus tail
Epito...
Epitop...
Epitope 3
C
1. SbSWEET4a
2. SbSWEET4b
3. SbSWEET4c
D
1. ZmSWEET4a
2. ZmSWEET4b
3. ZmSWEET4c

E

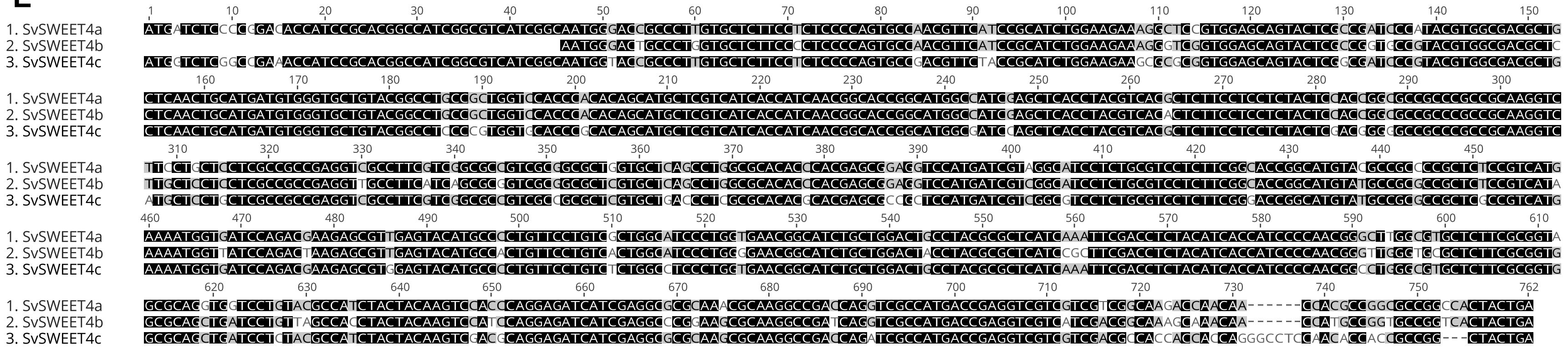

Fig. S5 Phylogeny and alignments of SWEET4 homologues.

Phylogenetic tree of SWEET4 homologues from *Setaria viridis*, *Sorghum bicolor*, *Zea mays* and *Oryza sativa* (A). Protein alignments of SWEET4a-c in *S. viridis* (B), *S. bicolor* (C) and *Z. mays* (D). CDS alignment of *SvSWEET4a-c* (E). Black denotes 100% sequence similarity.

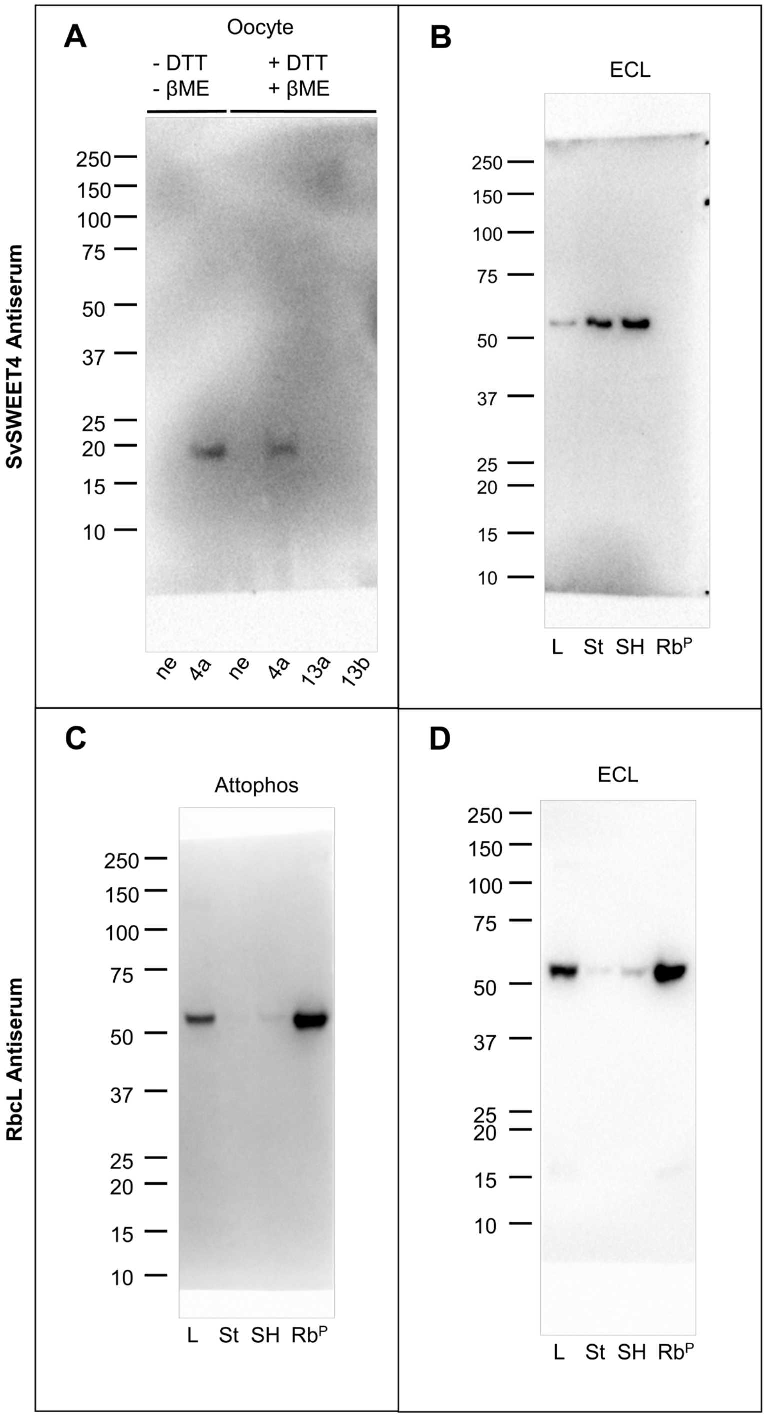

Fig. S6 Uncropped image of validation of SvSWEET4 antiserum using immunoblotting.

Commercially generated affinity-purified antiserum was raised against peptides derived from the SvSWEET4a protein isoform (see Figure S4). This was tested for specificity to SvSWEET4 against total protein extracts from Xenopus oocytes expressing SvSWEET4a, SvSWEETT13a or SvSWEET13b (A) and total plant protein extracts from

Setaria (B-D). In A the exclusion or addition (-/+) of reducing agents dithiothreitol (DTT) and β-mercaptoethanol (βME) is indicated above the blot. The lysates from non-expressing (ne), SvSWEET13a- and SvSWEET13b-expressing oocytes were used as negative controls. Recombinant protein was isolated and loaded from 20 Xenopus oocytes. Total plant extracts from the leaf (L); stem (St); and seed head (SH) were pooled from 12 Setaria plants harvested at 50% seed head emergence stage and 10 µg of protein was loaded. Purified Rubisco large subunit (Rb^P) from rice was used as a control and 4 µg was loaded. A, B and D used electrochemiluminescence (ECL) to detect the horseradish peroxidase (HRP) secondary antibody conjugate. C shows the membrane from B probed for the Rubisco large subunit (RbcL) using antiserum derived from tobacco RbcL peptides and the use of the AttoPhos substrate system to detect alkaline phosphatase (AP) secondary antibody conjugate. RbcL was probed for using a separate membrane in D using the same method as B. Molecular weight standards (kDa) are indicated to the left of each blot.

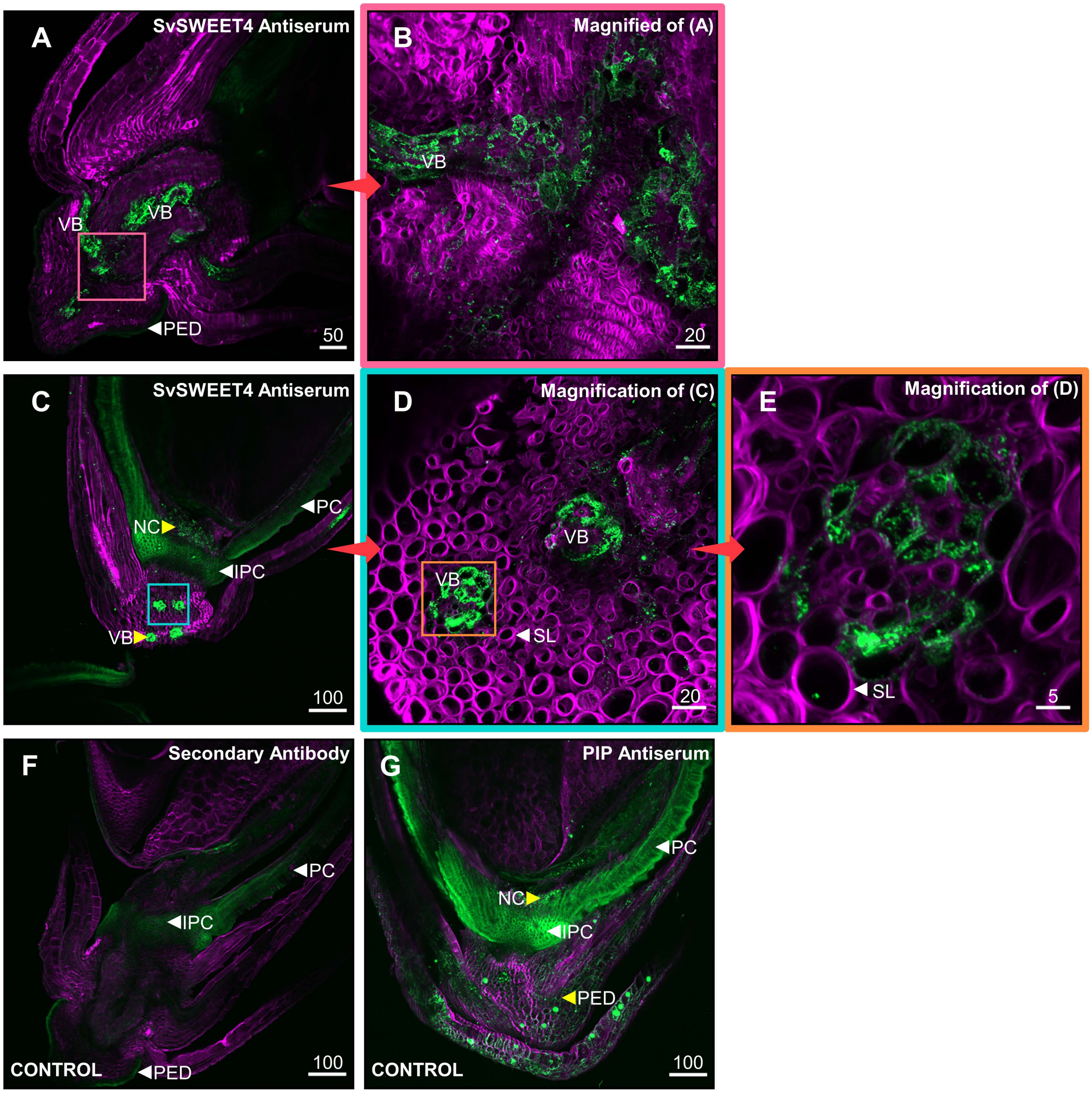

A
SvSWEET4 Antiserum
VB
VB
◀PED
50
B
Magnified of (A)
VB
20
C
SvSWEET4 Antiserum
◀PC
NC
◀IPC
VB
100
D
Magnification of (C)
VB
VB
◀SL
20
E
Magnification of (D)
◀SL
5
F
Secondary Antibody
◀PC
◀IPC
CONTROL
◀PED
100
G
PIP Antiserum
◀PC
NC
◀IPC
◀PED
CONTROL
100

Fig. S7 Immunolocalisation of SvSWEET4 to the vascular bundles of mature *Setaria viridis* (A.10) seeds.

Immunodetection of SvSWEET4 (A-E) to the vascular bundles (VB) was observed within the pedicel (PED). A and C shows two different sections of SvSWEET4 immunolabelling (yellow darts) to VBs. Micrograph B is a magnified region of A, with micrographs D and E being magnifications of C as indicated by arrows and coloured boxes. Autofluorescence of the PED; pericarp (PC) and inner pericarp (IPC) is indicated by white darts in A, C, F and G. Secondary antibody-only treated F and PIP G antiserum seed sections were used as controls. PIP was immunolabelled to the nucellus (NC) and PED, indicated by yellow darts in G. Sclerenchyma (SL). Fluorescence signals are pseudo-coloured: green - protein of interest labelled with secondary antibodies conjugated with Alexa Fluor 488 (excitation: 493 nm; emission: 517 nm); magenta - cell walls (excitation: 254 nm; emission: 432 nm). Scale bars represent microns (μM).

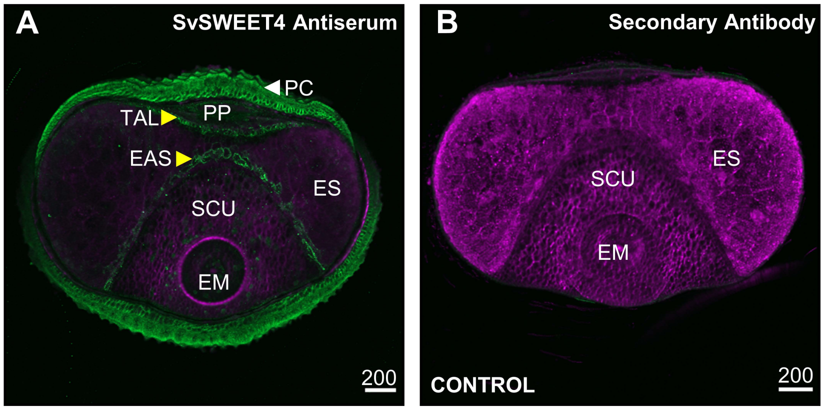

Fig. S8 Immunolocalisation of SvSWEET4 on transverse *Setaria viridis* (A.10) seeds.

Immunodetection of SvSWEET4 (A) was contrasted with secondary antibody-only treated (B) transverse seed sections. SvSWEET4 immunolabelled to the transfer aleurone (TAL) and endosperm adjacent to scutellum (EAS) layer as indicated by yellow darts. Autofluorescence of the pericarp (PC) is indicated by white darts in A. The PC was absent in B from the handling of seed sections, however, the autofluorescence is observed in the main image presented in Figure 5. Embryo (EM), scutellum (SCU), endosperm (ES). Fluorescence signals are pseudo-coloured: green - protein of interest labelled with secondary antibodies conjugated with Alexa Fluor 488 (excitation: 493 nm; emission: 517 nm); magenta - cell walls (excitation: 254 nm; emission: 432 nm). Scale bars represent microns (μM).

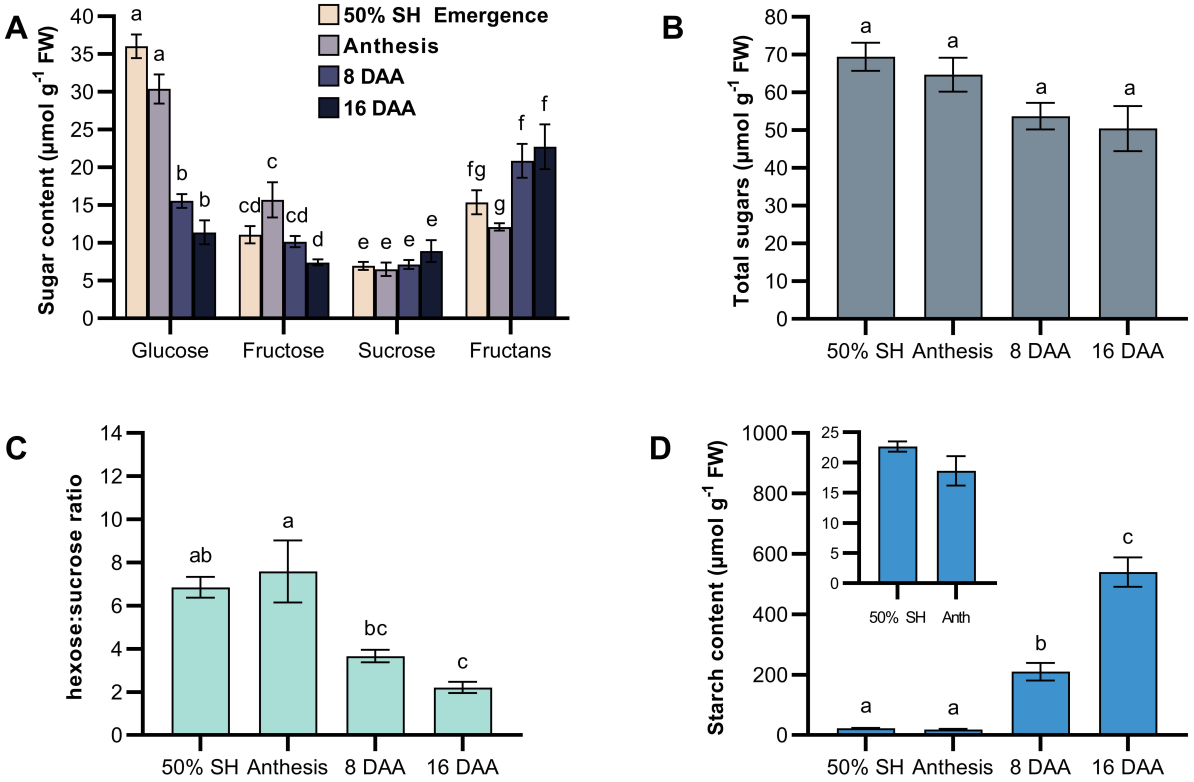

Fig. S9 Major carbohydrates of *Setaria viridis* (ME034V) seed heads at different developmental stages.

Plants harvested at 50% seed head emergence (50% SH), anthesis, 8 days after anthesis (DAA) and 16 DAA for carbohydrate analysis (A-D). Soluble sugars (A), total sugars (B), glucose:sucrose ratio (C) and starch (D) are displayed. For (d) the inset shows starch values at 50% SH emergence and anthesis that are not distinguishable. Bars represent the mean of four biological replicates, where each replicate was pooled from four plants. Error bars denote SEM. Letters denote significance (adjusted $p < 0.05$) between developmental stages for each carbohydrate as determined using a one-way ANOVA with Tukey's post-hoc for multiple comparisons.

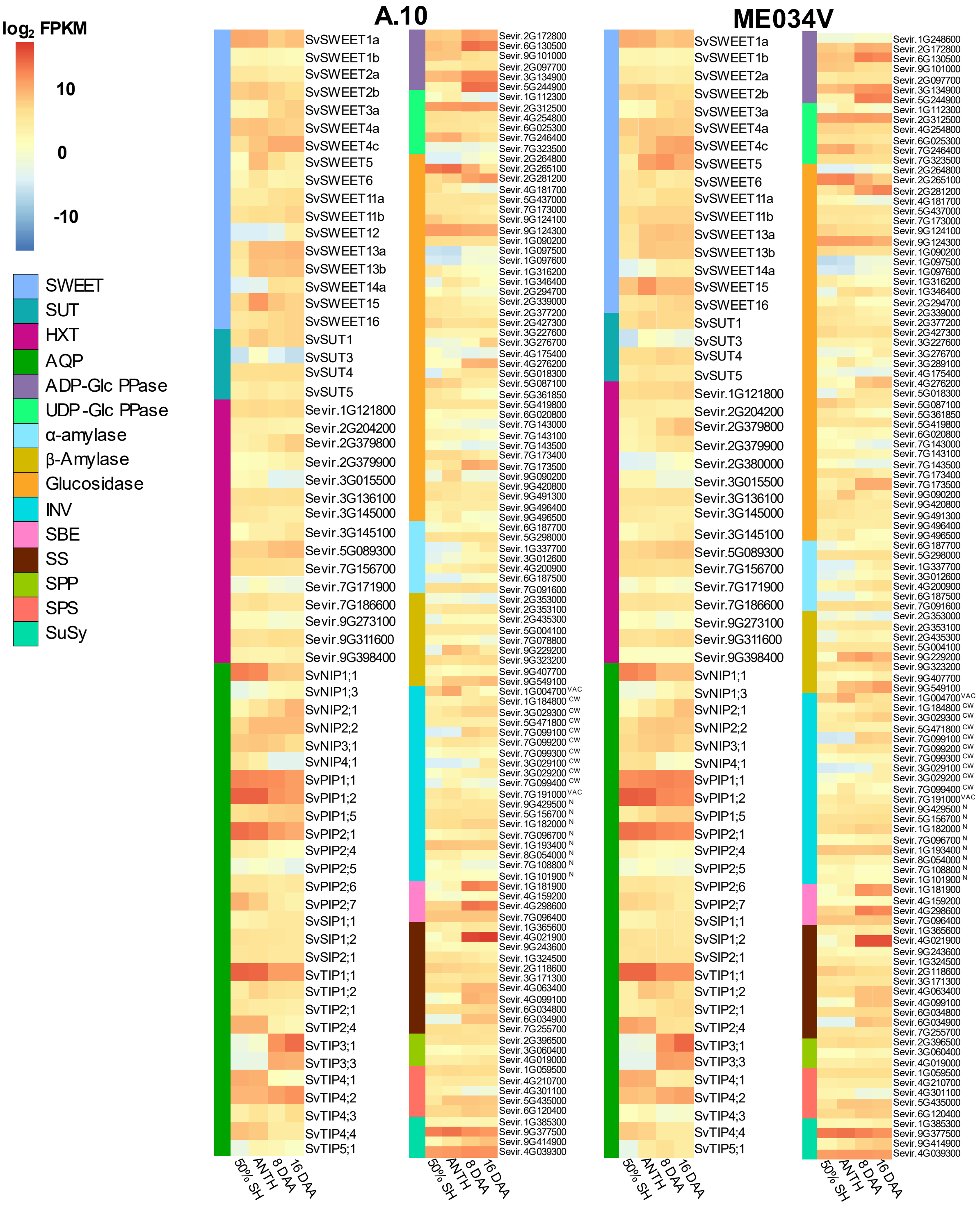

A.10
ME034V
log2 FPKM
10
0
-10
SWEET
SUT
HXT
AQP
ADP-Glc PPase
UDP-Glc PPase
α-amylase
β-Amylase
Glucosidase
INV
SBE
SS
SPP
SPS
SuSy
SvSWEET1a
SvSWEET1b
SvSWEET2a
SvSWEET2b
SvSWEET3a
SvSWEET4a
SvSWEET4c
SvSWEET5
SvSWEET6
SvSWEET11a
SvSWEET11b
SvSWEET12
SvSWEET13a
SvSWEET13b
SvSWEET14a
SvSWEET15
SvSWEET16
SvSUT1
SvSUT3
SvSUT4
SvSUT5
Sevir.1G121800
Sevir.2G204200
Sevir.2G379800
Sevir.2G379900
Sevir.2G380000
Sevir.3G015500
Sevir.3G136100
Sevir.3G145000
Sevir.3G145100
Sevir.5G089300
Sevir.7G156700
Sevir.7G171900
Sevir.7G186600
Sevir.9G273100
Sevir.9G311600
Sevir.9G398400
SvNIP1;1
SvNIP1;3
SvNIP2;1
SvNIP2;2
SvNIP3;1
SvNIP4;1
SvPIP1;1
SvPIP1;2
SvPIP1;5
SvPIP2;1
SvPIP2;4
SvPIP2;5
SvPIP2;6
SvPIP2;7
SvSIP1;1
SvSIP1;2
SvSIP2;1
SvTIP1;1
SvTIP1;2
SvTIP2;1
SvTIP2;4
SvTIP3;1
SvTIP3;3
SvTIP4;1
SvTIP4;2
SvTIP4;3
SvTIP4;4
SvTIP5;1
Sevir.1G248600
Sevir.2G172800
Sevir.6G130500
Sevir.9G101000
Sevir.2G097700
Sevir.3G134900
Sevir.5G244900
Sevir.1G112300
Sevir.2G312500
Sevir.4G254800
Sevir.6G025300
Sevir.7G246400
Sevir.7G323500
Sevir.2G264800
Sevir.2G265100
Sevir.2G281200
Sevir.4G181700
Sevir.5G437000
Sevir.7G173000
Sevir.9G124100
Sevir.9G124300
Sevir.1G090200
Sevir.1G097500
Sevir.1G097600
Sevir.1G316200
Sevir.1G346400
Sevir.2G294700
Sevir.2G339000
Sevir.2G377200
Sevir.2G427300
Sevir.3G227600
Sevir.3G276700
Sevir.3G289100
Sevir.4G175400
Sevir.4G276200
Sevir.5G018300
Sevir.5G087100
Sevir.5G361850
Sevir.5G419800
Sevir.6G020800
Sevir.7G143000
Sevir.7G143100
Sevir.7G143500
Sevir.7G173400
Sevir.7G173500
Sevir.9G090200
Sevir.9G420800
Sevir.9G491300
Sevir.9G496400
Sevir.9G496500
Sevir.6G187700
Sevir.5G298000
Sevir.1G337700
Sevir.3G012600
Sevir.4G200900
Sevir.6G187500
Sevir.7G091600
Sevir.2G353000
Sevir.2G353100
Sevir.2G435300
Sevir.5G004100
Sevir.7G078800
Sevir.9G229200
Sevir.9G323200
Sevir.9G407700
Sevir.9G549100
Sevir.1G004700 VAC
Sevir.1G184800 CW
Sevir.3G029300 CW
Sevir.5G471800 CW
Sevir.7G099100 CW
Sevir.7G099200 CW
Sevir.7G099300 CW
Sevir.3G029100 CW
Sevir.3G029200 CW
Sevir.7G099400 CW
Sevir.7G191000 VAC
Sevir.9G429500 N
Sevir.5G156700 N
Sevir.1G182000 N
Sevir.7G096700 N
Sevir.1G193400 N
Sevir.8G054000 N
Sevir.7G108800 N
Sevir.1G101900 N
Sevir.1G181900
Sevir.4G159200
Sevir.4G298600
Sevir.7G096400
Sevir.1G365600
Sevir.4G021900
Sevir.9G243600
Sevir.1G324500
Sevir.2G118600
Sevir.3G171300
Sevir.4G063400
Sevir.4G099100
Sevir.6G034800
Sevir.6G034900
Sevir.7G255700
Sevir.2G396500
Sevir.3G060400
Sevir.4G019000
Sevir.1G059500
Sevir.4G210700
Sevir.4G301100
Sevir.5G435000
Sevir.6G120400
Sevir.1G385300
Sevir.9G377500
Sevir.9G414900
Sevir.4G039300
50% SH
ANTH
8DAA
16DAA

Fig. S10 Expression of genes encoding sugar transporters, aquaporins and enzymes involved in sugar metabolism within *Setaria viridis* seed heads.

Heatmaps displaying log_2 FPKM values of select genes encoding proteins involved in sugar transport or metabolism in seed heads of *Setaria viridis* ecotypes A.10 and ME034V. Whole seed heads were harvested at four stages of development: 50% seed head emergence, anthesis (ANTH), 8 days after anthesis (DAA) and 16 DAA. Values represent the mean from four biological replicates where each replicate is pooled from four plants. Row colours denote gene families. The different types of invertases are indicated beside their gene IDs: neutral (N), cell wall (CW) and vacuolar (VAC). Sucrose transporter (SUT), hexose transporter (HXT), aquaporin (AQP), ADP- and UDP-glucose pyrophosphorylase (ADP- and UDP-Glc PPase), invertase (INV), starch branching enzyme (SBE), starch synthase (SS), sucrose phosphatase (SPP), sucrose phosphate synthase (SPS) and sucrose synthase (SuSy).

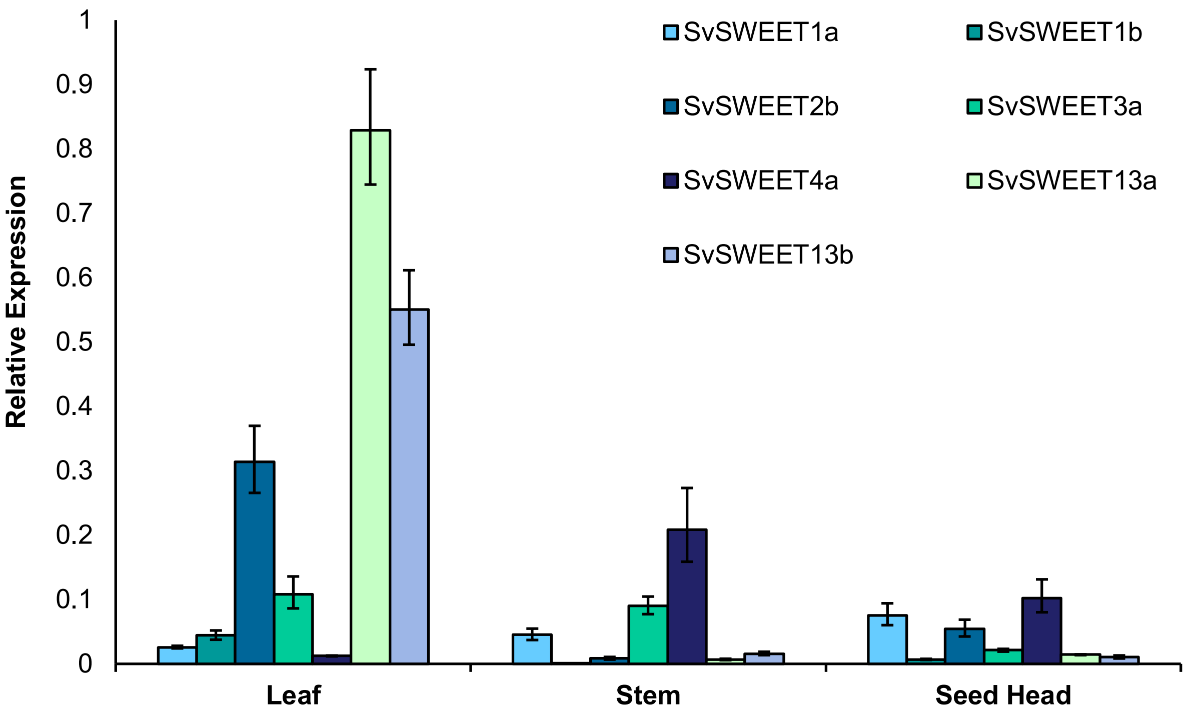

Fig. S11 Relative expression of a subset of *Setaria viridis* SWEETs using qPCR.

Relative expression of *SvSWEET1a*, *1b*, *2b*, *3a*, *4a*, *13a* and *13b* was measured compared to geometric means of reference genes *elongation factor 1-α* and *translation factor*. Leaf, stem and seed head tissues were harvested from Setaria plants at 50% seed head emergence. Bars represent the mean from four biological replicates where each replicate was pooled from three plants. Error bars represent SEM.

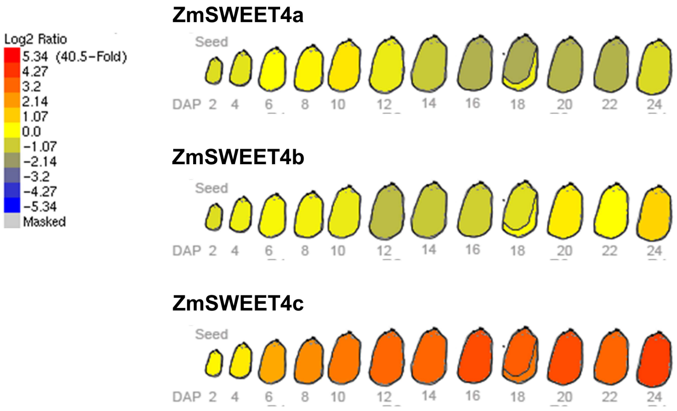

Fig. S12 Electronic fluorescent pictograph (eFP) of ZmSWEET4 orthologues.

The eFP depicts relative expression (log_2 ratio) of *ZmSWEET4a*, *ZmSWEET4b* and *ZmSWEET4c* in the maize seed development across days after pollination (DAP). This eFP was generated from http://bar.utoronto.ca/ (Winter *et al.*, 2007; Sekhon *et al.*, 2011).

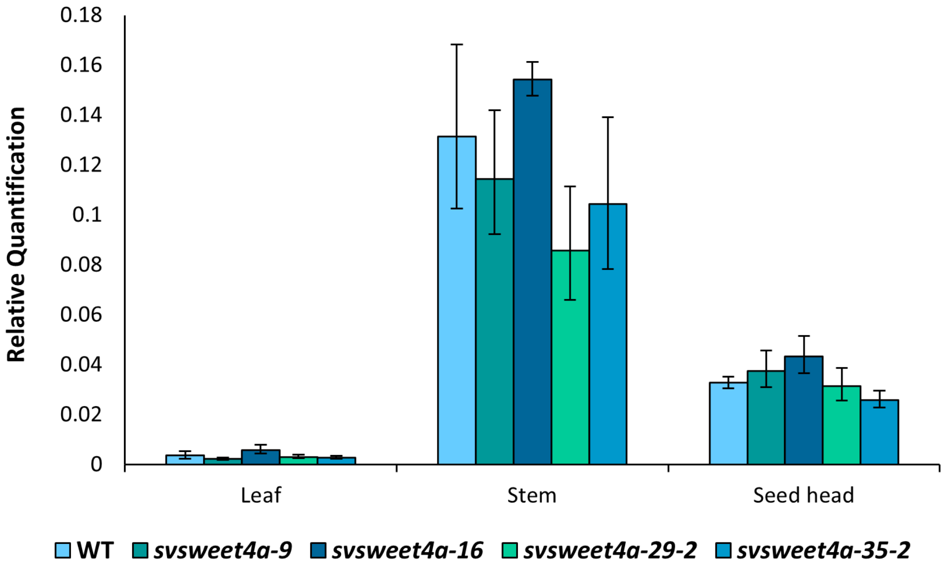

Figure S13 Expression analysis of *SvSWEET4a* targeted by RNAi in T2 *Setaria viridis* plants.

Expression analysis of SWEET genes targeted by RNAi in T2 *S. viridis* plants. The youngest fully expanded leaf, elongating internode of stem and whole seed head samples were harvested at 50% seed head emergence. Mean ± SEM, n = 3-4. Relative transcript levels of SvSWEET4a between wild type (WT) and *svsweet4a* knockdown lines. No significant reduction in target SWEET transcript abundances were observed in other lines compared to the WT.

Table S1 List of primers used for qPCR of reference genes and *SvSWEETs*.

Gene	Forward Primer 5′ - 3′	Reverse Primer 5′ - 3′
SvSWEET1a	CCGCGACCCCTTCATCATTAT	CCCTTGTTCTTCCGGTACATGG
SvSWEET1b	TCGCCGCTCTCCATCATG	CGAGCAGGCCGTAGACAAA
SvSWEET2b	GTCCCTCTCCACCTTCCTCA	GCATTGCTCCCAGGATGACT
SvSWEET3a	TGCCTTTCTACTTGTCGCTGT	GTTAAACACCCGACGCTGTT
SvSWEET4a	CGCGCTCATCAAATTCGACC	GTAGATGGCGTACAGGACCAC
SvSWEET13a	TCACGCTGGGCTGGATCT	AGAGGGAGAGGGAGAATGGC
SvSWEET13b	GGGTGGCCTAGACGACTCAA	CGCCATCGTTGCTCATGAC
Elongation Factor 1-α (Sevir.3G271600)	CCACACCTCCCACATAGCTG	CCAGCATCACCGTTCTTCAGG
Translation Factor (Sevir.5G315700)	CAATGTGAAAGCGAAGATCCAGG	CCTTCTGAATGTTGTAGTCTGCC

References

Sekhon RS, Lin H, Childs KL, Hansey CN, Robin Buell C, De Leon N, Kaeppler SM. 2011. Genome-wide atlas of transcription during maize development. The Plant Journal **66**, 553–563.

Winter D, Vinegar B, Nahal H, Ammar R, Wilson G V., Provart NJ. 2007. An "Electronic Fluorescent Pictograph" browser for exploring and analyzing large-scale biological data sets (I Baxter, Ed.). PLoS ONE **2**, e718.
